# Potential compensatory mechanisms preserving cardiac function in myotubular myopathy

**DOI:** 10.1007/s00018-024-05512-9

**Published:** 2024-12-03

**Authors:** Alix Simon, Nadège Diedhiou, David Reiss, Marie Goret, Erwan Grandgirard, Jocelyn Laporte

**Affiliations:** Institute of Genetics and Molecular and Cellular Biology (IGBMC), INSERM U1258, CNRS UMR7104, University of Strasbourg, 1 rue Laurent Fries, Illkirch, 67404 France

**Keywords:** Centronuclear myopathy, Myotubularin, Dynamin, Phosphoinositides, Omics, Integrin

## Abstract

X-Linked myotubular myopathy (XLMTM) is characterized by severe skeletal muscle weakness and reduced life expectancy. The pathomechanism and the impact of non-muscular defects affecting survival, such as liver dysfunction, are poorly understood. Here, we investigated organ-specific effects of XLMTM using the *Mtm1*^−/y^ mouse model. We performed RNA-sequencing to identify a common mechanism in different skeletal muscles, and to explore potential phenotypes and compensatory mechanisms in the heart and the liver. The cardiac and hepatic function and structural integrity were assessed both in vivo and in vitro. Our findings revealed no defects in liver function or morphology. A disease signature common to several skeletal muscles highlighted dysregulation of muscle development, inflammation, cell adhesion and oxidative phosphorylation as key pathomechanisms. The heart displayed only mild functional alterations without obvious structural defects. Transcriptomic analyses revealed an opposite dysregulation of mitochondrial function, cell adhesion and beta integrin trafficking pathways in cardiac muscle compared to skeletal muscles. Despite this dysregulation, biochemical and cellular experiments demonstrated that these pathways were strongly affected in skeletal muscle and normal in cardiac muscle. Moreover, biomarkers reflecting the molecular activity of MTM1, such as PtdIns3*P* and dynamin 2 levels, were increased in the skeletal muscles but not in cardiac muscle. Overall, these data suggest a compensatory mechanism preserving cardiac function, pointing to potential therapeutic targets to cure the severe skeletal muscle defects in XLMTM.

**Supplementary Information:**

The online version contains supplementary material available at 10.1007/s00018-024-05512-9.

## Introduction

X-Linked Centronuclear Myopathy (XLMTM), also called myotubular myopathy (MIM#310400), is associated with very severe muscle weakness and reduced life expectancy. XLMTM is caused by a loss-of-function of *MTM1*, a gene located on the X chromosome. *MTM1* is ubiquitously expressed in humans and mice with no obvious tissue-specific splice isoforms [1], and codes for the lipid phosphatase myotubularin that dephosphorylates phosphatidylinositol 3-phosphate (PtdIns3*P*). Myotubularin regulates membrane structures such as triads in skeletal muscle, membrane trafficking and integrin recycling [2–4]. Patients with XLMTM display generalized muscle weakness and hypotrophy, and hypotonia at birth. More than half of patients die by two years of age [5, 6]. They also experience severe respiratory failure and most require ventilator assistance. Their muscle phenotype is linked to specific histological hallmarks such as centralization of nuclei and other organelles including mitochondria, and hypotrophic myofibers with type I (slow) predominance [7, 8]. Female heterozygous carriers may develop similar albeit usually milder symptoms compatible with normal lifespan [9–11].

The implication of non-muscle phenotypes in the disease and survival remains unclear. However, several non-muscle phenotypes were associated to XLMTM, in particular in long-term survivors [12]. Female carriers often display skeletal asymmetries while male patients have an elongated face and are taller than normal at birth [9, 10, 13]. Liver dysfunction is recently becoming a concern for XLMTM patients, as several patients died after hepatic peliosis (liver hemorrhage) [12, 14, 15] while others display hypertransaminemia, hyperbilirubinemia and intrahepatic cholestasis [16, 17]. Moreover, a recent gene therapy clinical trial using adeno-associated viral (AAV) transduction of MTM1 in XLMTM males was halted due to hepatic failure leading to the death of 4 patients. It is thus possible that AAV-MTM1 injection exacerbated an underlying hepatic defect [18]. While no study of the liver function has been performed in the *Mtm1*^*−/y*^ mouse, hepatic defects were shown recently in the zebrafish *mtm* model [19]. Concerning cardiac muscle, there were no consistent phenotypes reported so far in XLMTM patients or carriers, with only 10–15% of patients presenting conduction or heart rhythm abnormalities in a prospective international natural history study [20]. In XLMTM dogs, cardiac echography revealed abnormalities in diastolic function, with no histological defects [21].

To study this disease, a murine model based on the knock-out of *Mtm1* was created. *Mtm1*^−/y^ mice faithfully replicate the phenotypes of patients, as they develop a severe muscle weakness and hypotrophy from 2 to 3 weeks which leads to death by about 8 weeks, and the typical histopathological hallmarks can be observed [22]. Transcriptomic studies of the *Mtm1*^−/y^ murine model have previously been performed on single muscles [23–25]. They have led to the identification of differentially expressed genes and key altered pathways such as muscle contraction, regeneration, and inflammation. However, the molecular characterization of only one skeletal muscle limits our understanding of the disease. Thus, the pathological mechanisms remain poorly understood in skeletal muscle and the potential non-muscle defects are yet to be characterized. Tackling these bottlenecks is of importance to identify therapeutic targets as there is no treatment to date.

This study aims to investigate organ-specific effects of XLMTM. We performed RNA-sequencing on previously uncharacterized skeletal muscles such as gastrocnemius and diaphragm from wild-type (WT) and *Mtm1*^−/y^ mice to better define a common pathological alteration common to different muscles. Heart and liver were similarly studied by RNA-seq and the cardiac and hepatic functions were studied with in vivo functional phenotyping, histology, electron microscopy, immunofluorescence labeling, and biochemical assays. We identified a common disease signature across skeletal muscles, and an opposite pattern of dysregulation in the heart suggesting a compensatory mechanism.

## Materials and methods

### Animals

The *Mtm1* knock-out mouse model (*Mtm1*^−/y^) and the wild-type (WT) littermates bred on a pure 129Pas background were used for experiments. The *Mtm1*^−/y^ mouse line was generated and identified through PCR genotyping from mouse tail DNA as previously described [22]. Mice were placed in ventilated cages with free access to food and water in temperature-controlled rooms with 12 h daylight/dark cycles.

### Sample collection and histology

All mice analyzed in this study were male as the *Mtm1* gene is on the X chromosome and the disease is recessive. Heart, liver, diaphragm, and gastrocnemius were dissected and snap-frozen in liquid nitrogen at 5 weeks for RNA-seq. For western blotting and/or ELISA assay, heart, liver, diaphragm, gastrocnemius and tibialis anterior (TA) samples were dissected at 7 weeks and snap-frozen in liquid nitrogen. For haematoxylin and eosin (H&E) histology analysis, samples were collected at 7 weeks for the *Mtm1*^−/y^ and WT males, at 8 months for *Mtm1*^+/−^ and WT females. Then, they were frozen in liquid nitrogen-cooled isopentane and stored at −80 °C. Transversal cryosections (8 μm) were prepared and stained and were observed using the Hamamatsu 322 NanoZoomer 2HT slide-scanner. Blood was collected in heparin-coated tubes and plasma was stored at −80 °C.

### RNA extraction and RNA-sequencing

RNA was extracted from whole heart, liver, diaphragm and gastrocnemius samples using TRI Reagent (Molecular Research Center, Cincinnati, OH, USA), and precipitated in a mix of sodium acetate (0.1 vol) and ethanol (2.5 vol). RNA-Seq libraries were generated from 350 ng of total RNA using TruSeq Stranded mRNA Library Prep Kit and TruSeq RNA Single Indexes kits A and B (Illumina, San Diego, CA), according to manufacturer’s instructions. cDNA libraries were checked for quality and quantified using capillary electrophoresis. Sequencing was performed with Illumina HiSeq 4000 as single-end 50-bp reads.

### Transcriptome analysis

For heart, liver, diaphragm and gastrocnemius samples, reads were preprocessed in order to remove adapter, polyA and low-quality sequences (Phred quality score below 20). After this preprocessing, reads shorter than 40 bases were discarded from further analysis. These preprocessing steps were performed using cutadapt version 1.10 [26]. Reads were mapped onto the mm10 assembly of Mus musculus genome using STAR version 2.5.3a [27]. Gene expression quantification was performed from uniquely aligned reads using htseq-count version 0.6.1p1 [28], with annotations from Ensembl version 96 and “union” mode. Only non-ambiguously assigned reads have been retained for further analyses. For tibialis anterior samples, openly available gene expression quantification data from a previous study were downloaded from NCBI GEO: GSE160084 [23]. To compare our results with transcriptomic datasets obtained from C57BL/6J *Mtm1*^−/y^ mice, tibialis anterior and quadriceps datasets were downloaded from NCBI GEO: GSE207447 [24] and GSE120336 [25], and batch-effect correction was performed with the limma package [29].

The number of samples used for transcriptome analysis was as follows: 3 mice for the WT liver and *Mtm1*^−/y^ diaphragm samples, 5 mice for *Mtm1*^−/y^ tibialis anterior at 2 and 7 weeks and WT tibialis anterior samples at 7 weeks, 4 mice for all other groups. Count tables were analyzed in the open-source RStudio environment for R (4.1.0). The DESeq2 package (version 1.34.0) [30] was used to normalize, fit, and compare the data between groups. Cutoff values for differentially expressed gene determination were as follows: Benjamini-Hochberg adjusted *P* value < 0.05 and absolute value of log2FC > 1.

### Electron microscopy

The samples were fixed by immersion in 2.5% glutaraldehyde and 2.5% paraformaldehyde in cacodylate buffer (0.1 M, pH 7.4), and washed in cacodylate buffer for further 30 min. The samples were postfixed in 1% osmium tetroxide in 0.1 M cacodylate buffer for 1 h at 4 °C and dehydrated through graded alcohol (50, 70, 90 and 100%) and propylene oxide for 30 min each. Samples were oriented and embedded in Epon 812. Semithin sections were cut at 2 μm and ultrathin sections were cut at 70 nm (Leica Ultracut UCT) and contrasted with uranyl acetate and lead citrate and examined at 70 kV with a Morgagni 268D electron microscope (FEI Electron Optics) equipped with a Mega View III camera (Soft Imaging System) or with a Philips CM12 electron microscope equipped with a Gatan OneView Camera (Gatan).

### Non-invasive blood pressure (NIBP)

Systolic blood pressure and heart rate were determined in conscious mice with the tail-cuff method using the BP-2000 Blood Pressure Analysis System (Visitech Systems).

### Electrocardiography

To record the ECG, mice were anaesthetized with 1–2% of isoflurane in 700 ml O_2_/minute via facemask (following induction in a chamber containing 5% isoflurane). Rectal temperature was continuously monitored and maintained within 37–38 °C using a heat pad and heat lamp. The lead II ECG was recorded from needle electrodes inserted subcutaneously into the right forelimb and into each hindlimb. The signal was acquired for up to 120s using Chart 4.2.3 software, an electrocardiograph ISO DAM8 amplifier (World Precision Instruments, USA) and analogic-numeric conversion box (ITF16A/D converter, EMKA technologies, USA). For each cardiac cycle, the P, Q, R, S and T peaks were defined and used to derive the parameters shown in Table 1.

**Table 1:** Non-invasive blood pressure data

	WT	Mtm1^−/y^	*P* value	Significance
Heart rate (bpm)	620 ± 10	585 ± 23	0.19	ns
Systolic blood pressure (mmHg)	108 ± 3	113 ± 4	0.27	ns

Values are mean ± SEM. *n* = 8 mice in each group. ns: not statistically significant (*P* value > 0.05)

### Echocardiography

Transthoracic Echocardiographic images were captured using a VisualSonics Vevo 2100 Imaging System (Toronto, Canada) with a MS400 probe (30-MHz). The echographers allow M-mode, B-mode, and Doppler imaging with ECG and respiratory gating of all images. The thorax was treated by mechanic shaving and hypoallergenic cream hair remover (NairT^M^) to optimize the acoustic interface. Mice were anesthetized using 1–2% isoflurane in oxygen and positioned supine on the heating pad of the Vevo system, which maintains normo thermia by continuous monitoring of the rectal temperature. Prewarmed ultrasound gel was applied on the thorax. The VisualSonics rail system was used to fix the probe, avoiding any compression of the thorax. The cardiac morphology and ventricular systolic function were acquired by M-mode tracings of the left ventricle (LV) using the short-axis view, with the ultrasound beam perpendicular to the LV at the midpapillary level to determine ejection fraction (EF), fractional shortening (FS), wall thickness, LV inner diameter (LVID), and LV mass (LVM = 1.055x [(EDD + SW + PW)^3^-EDD^3^)]). Aortic dimensions were determined using B-mode imaging in the parasternal long-axis views. Pulmonary and aortic artery velocity and pressures were evaluated using color Doppler imaging in pulmonary artery and aortic arch to detect intra-cardiac pressures changes (aortic valve and right ventricle (RV) function). Stroke volume (SV) and cardiac output (CO) were calculated. Diastolic function was assessed by color Doppler mode from the apical 4 chamber view for mitral valve (MV) and tricuspid valve (TV) to detect left and right diastolic filling patterns. The MV measurements performed were the following: MV early wave peak (MVE), MV atrial wave peak (MVA), aortic ejection time (AET), isovolumic relaxation time (IVRT), isovolumic contraction time (IVCT). The TV measurements included TV early wave peak (TVE) and TV atrial wave peak (TVA). The TV velocity time integral (VTI) and TVE/A ratio were calculated. Images were captured on cine loops at the time of the study, and measurements were done off-line using Vevo Lab analysis software (version 3.3.5). The statistical analysis was obtained by Graph Pad (version 8.0.3).

### Blood chemistry

Blood chemistry was performed on an OLYMPUS AU-480 automated laboratory work station (Beckmann Coulter, US) with kits and controls supplied by Beckmann Coulter.

### Tissue immunolabeling

TA muscles and hearts were frozen in liquid nitrogen-cooled isopentane and stored at -80 °C for β1-integrin staining. Transversal cryosections (8 μm) were prepared. Livers were fixed in paraformaldehyde (PFA) and embedded in paraffin. Paraffin Sect. (5 μm) were prepared. TA muscle and heart cryosections were thaw and washed three times in PBS. Liver sections were treated for deparaffinization with xylene, washed with ethanol and hydrated in a series of graded alcohols until water was used. The sections were permeabilized with 0.2% PBS-Triton X-100 for 10 min and saturated with 5% BSA in 0.1% PBS-Triton X-100 for 1 h. Antibodies were diluted in 0.1% PBS-Triton X-100 5% BSA and incubation was performed at room temperature for 1h30min for the primaries and 1 h for the secondaries. Primary antibodies were rat anti-integrin β1 MAB1997 (Millipore) 1:100, BSEP polyclonal PA5-78690 (Invitrogen) 1 µg/ml, mouse monoclonal embryonic myosin-3 (BF-45, DHSB) 1:200, and rabbit polyclonal laminin gamma 3 antibody (ab11575) 1:200. Secondary antibodies were respectively Alexa-Fluor 488 goat anti-rat IgG, Alexa-Fluor 488 goat anti-rabbit IgG, Alexa-Fluor 488 donkey anti-mouse IgG, and Alexa-Fluor 594 donkey anti-rabbit IgG (Invitrogen). Images were acquired using the Leica DM 4000 B upright motorized microscope or Zeiss Axio Observer Z1. Quantification of fibers with integrin β1 abnormal localization was done manually and over 300 fibers were counted per sample. Quantification of embryonic myosin and laminin signal intensity was performed automatically with custom ImageJ macros (see below).

### DHE ROS staining

TA muscles, gastrocnemius and hearts were frozen in liquid nitrogen-cooled isopentane and stored at -80 °C for DHE staining. Transversal cryosections (8 μm) were prepared. Cryosections were thawed and washed in PBS. The sections were incubated for 30 min at 37 °C with 2.5 µM Dihydroethidium (DHE) D23107, Invitrogen 1:1000 in phosphate-buffered saline. DHE produces a red fluorescence when oxidized to ethidium bromide by ROS. The sections were washed in PBS and nucleus and membranes were stained for 15 min at room temperature respectively with DAPI and wheat germ agglutinin (WGA). After staining the sections were rinsed, air-dried, mounted in ProLong Gold antifade reagent (P36934, Invitrogen), and the emission signal was recorded within two hours using the Zeiss Axio Observer Z1 microscope. Quantification of ROS signal intensity was performed automatically for all fibers with a custom ImageJ macro (see below).

### Analysis of ROS staining, embryonic myosin and laminin

Image analysis was conducted using custom ImageJ macros. Briefly, fibers were segmented using Cellpose [31], followed by quantification of DHE ROS staining and embryonic myosin signal intensity within each fiber. To assess extracellular matrix thickness, a 2 μm-wide region of interest was defined around each fiber, capturing the pericellular laminin signal. The average laminin signal intensity within this region was calculated for each fiber. As a result, fibers with thinner laminin signal showed lower mean intensity values. The data for all fibers were averaged for each biological replicate.

### RNA extraction and RT-qPCR analysis

Total RNA was isolated from mouse TA, gastrocnemius, diaphragm and heart using TRI Reagent (MRC). cDNA was synthesised from 1 µg of RNA by reverse transcription using Transcriptase inverse Transcriptor (Roche, 03531287001), according to the manufacturer’s protocol. Quantitative RT-PCR analysis was performed with gene-specific primers using the LightCycler 480 SYBR Green I Master (Roche, 04887352001), according to the manufacturer’s protocol. For each sample, the relative abundance of the transcript level of a given gene was calculated by normalization to a housekeeping gene (Rpl27). RT-qPCR primers are provided in Supplementary Dataset S15.

### Protein extraction and western blotting

Heart, diaphragm, TA and gastrocnemius were lysed in RIPA buffer supplemented with 1 mM PMSF, 1 M DTT and complete mini EDTA-free protease inhibitor cocktail (Roche Diagnostic). Protein concentrations were determined with the Pierce BCA Protein Assay Kit (Thermo Fisher scientific). 5x Pierce Lane Marker Non-Reducing Sample Buffer (Thermo Fisher scientific) was added to the samples and denaturation was performed for 5 min at 95 °C. Then 15 µg of protein were separated in 10% SDS-PAGE gel and transferred on nitrocellulose membrane for 7 min at 2.5 A using a Trans-Blot Turbo Transfer System (Bio-Rad). Total protein was determined by Ponceau S staining. Membranes were blocked for 1 h in TBS containing 5% non-fat dry milk and 0.1% Tween20 before incubation 1h30min with primary antibodies diluted in blocking buffer containing 5% milk. Secondary antibodies coupled to a horseradish peroxidase were incubated for 1 h. Primary antibodies used were SOD2 (D9V9C) 13,194 (Cell Signaling) 1:1000, and rabbit polyclonal against DNM2 2865 1:500 previously described [31]. Secondary antibody was goat anti-rabbit (Jackson Immunoresearch) 1:10 000. Nitrocellulose membranes were visualized in Amersham Imager 600 (GE Healthcare Life Sciences). ImageJ was used to quantify bands intensity. Data were normalized to Ponceau red staining and the mean value of control group.

### ELISA assay

PtdIns3*P* levels were determined by using the PI3P Mass ELISA Kit (Catalog #K-3300, Echelon Biosciences, USA) according to the manufacturer’s instructions.

### Data representation and statistical analyses

**Fig. 1:**
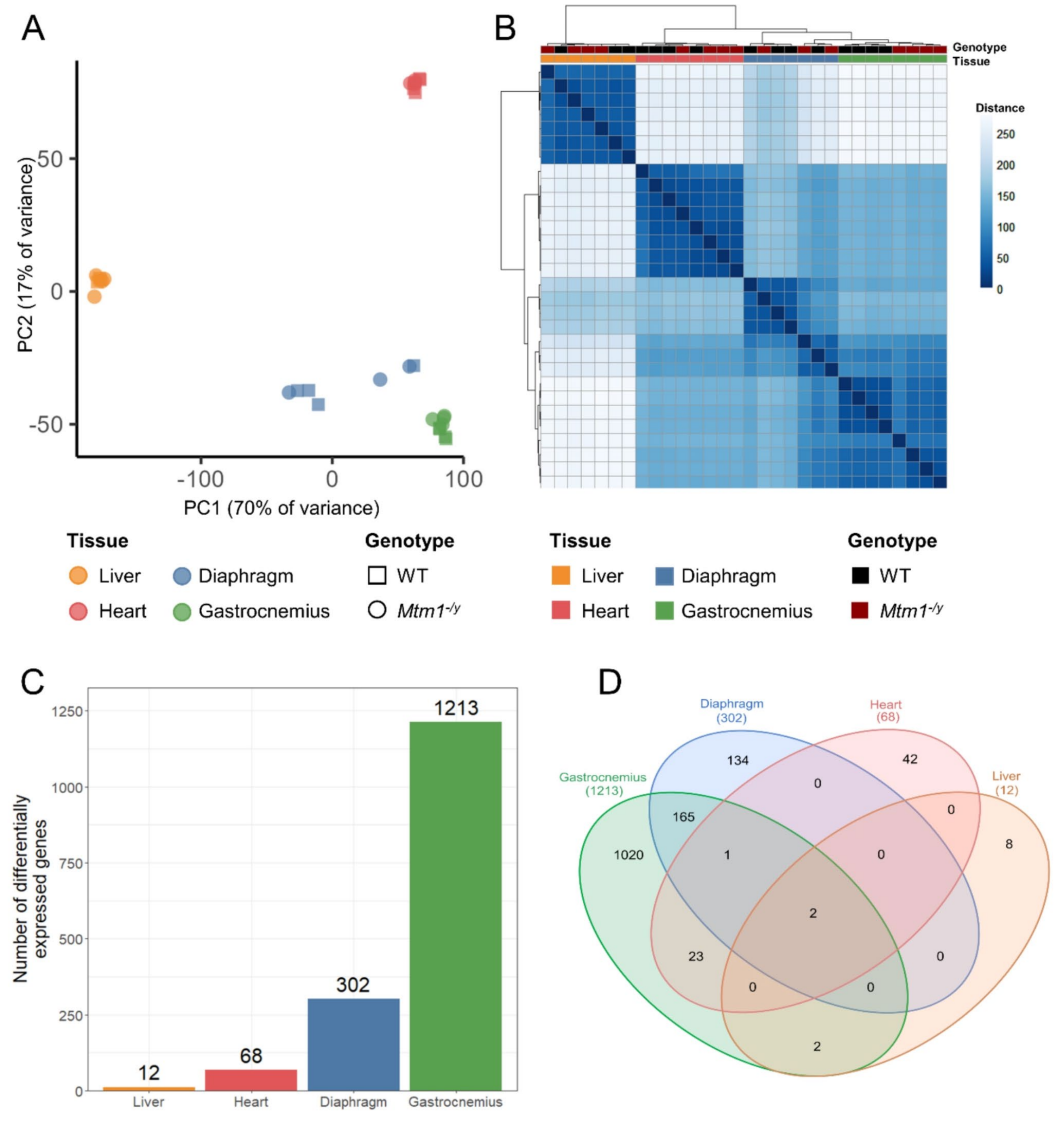
The effect of XLMTM on gene expression is highly organ-specific. Exploratory data analysis and differential gene expression analysis of RNA-seq data of liver, heart, diaphragm and gastrocnemius samples taken from WT and *Mtm1*^−/y^ mice at 5 weeks. **A** Principal component analysis, principal components 1 and 2 are shown. **B** Unsupervised hierarchical clustering based on sample-to-sample similarity. **C** Bar plot showing the number of DEGs between WT and *Mtm1*^−/y^ samples for each tissue. **D** Venn diagram illustrating the number of common and specific DEGs across tissues

PCA plots, volcano plots, bar plots, and enrichment analyses results were generated in R-Bioconductor (R 4.1.0) with the ggplot2 package (version 3.4.2) [32]. PCA plots (Figs. 1A and 3A) were generated from the normalized counts of all genes with variance-stabilizing transformation. Hierarchical clustering (Fig. 1B) was performed on Euclidean distance between samples, using Ward’s hierarchical agglomerative clustering method [33]. Venn diagram in Fig. 1D was obtained from the InteractiVenn website (http://www.interactivenn.net). The proportional Venn diagram (Fig. 3C) was created using the eulerr package (version 7.0.0) [34]. Over-representation analysis (Fig. 3D) was performed using the ClusterProfiler package (version 4.2.2) [35], and GO terms with a Benjamini-Hochberg (BH) adjusted *P* value < 0.05 were defined as enriched. Gene set enrichment analyses (GSEA) were performed on signal-to-noise ratio (SNR) ranked genes with the fgsea package (1.20.0) [36] using hallmarks, Reactome pathways, and gene ontology (GO) gene sets downloaded from the Molecular Signature Database (MSigDb) mouse collections [37–40], and gene sets with a BH adjusted *P* value < 0.05 were defined as enriched (Figs. 3E and 5D and Supplementary Figures S1, S3 and S4). The full R code, and data used for the analysis are available on Zenodo: 10.5281/zenodo.14184555. Western blot and ELISA results were analyzed in GraphPad Prism (v9) using a Student’s t-test.

**Fig. 2:**
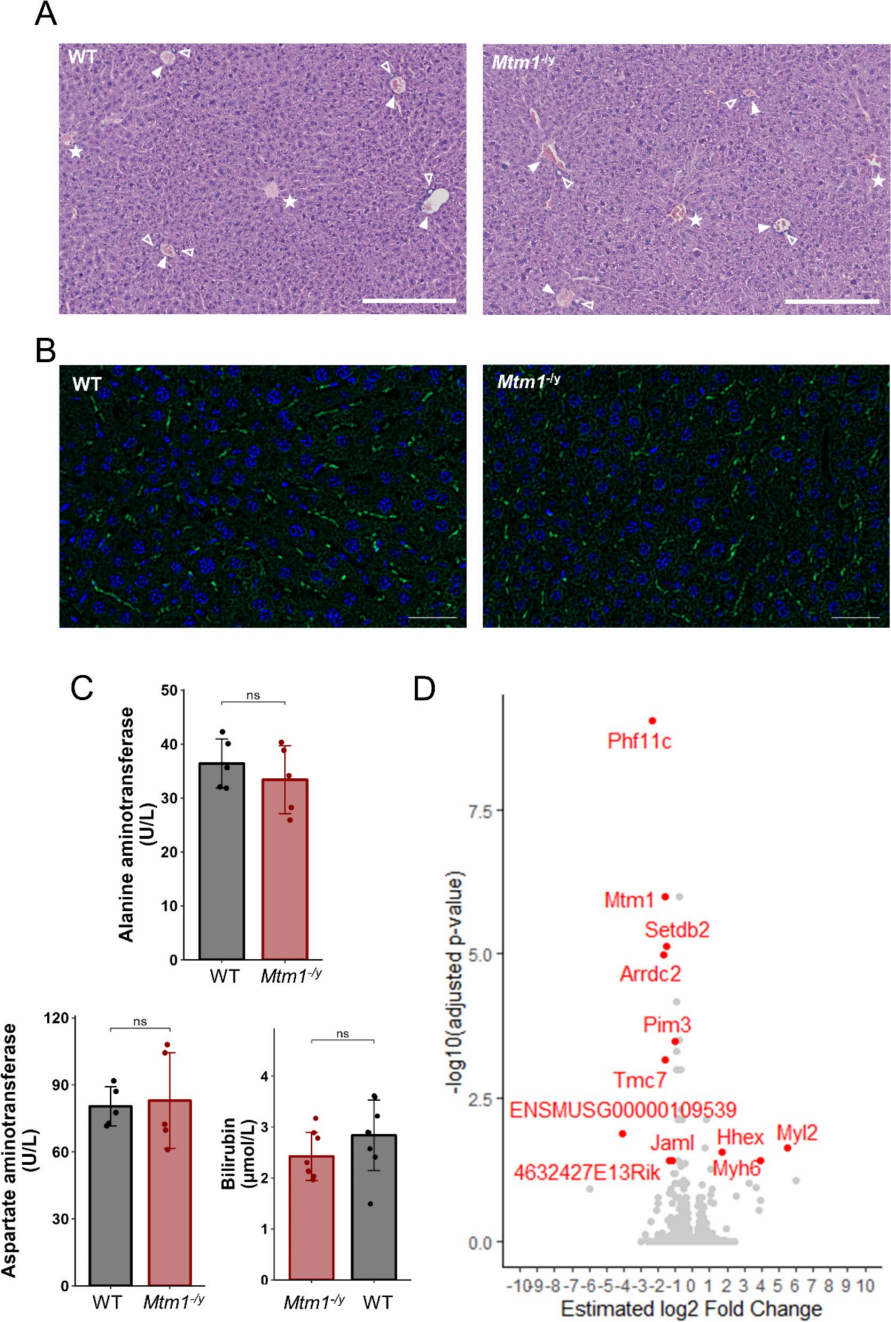
The *Mtm1*^−/y^ mouse model shows no liver defects. **A** Representative liver histology of WT and *Mtm1*^−/y^ mice (H&E). Stars indicate central veins, full arrows indicate portal veins, and empty arrows indicate bile ducts. Scale bars 250 μm. **B** Representative immunofluorescent staining images of BSEP (green) and nuclei (DAPI, blue) in liver of WT and *Mtm1*^−/y^ mice. Scale bars 50 μm. **C** Bar charts showing levels of alanine aminotransferase (top) and aspartate aminotransferase (bottom left) and bilirubin (bottom right) in serum of WT and *Mtm1*^−/y^ mice. Student’s t-test: ns: *P* > 0.05. **D** Volcano plot showing the 12 DEGs in liver

**Fig. 3:**
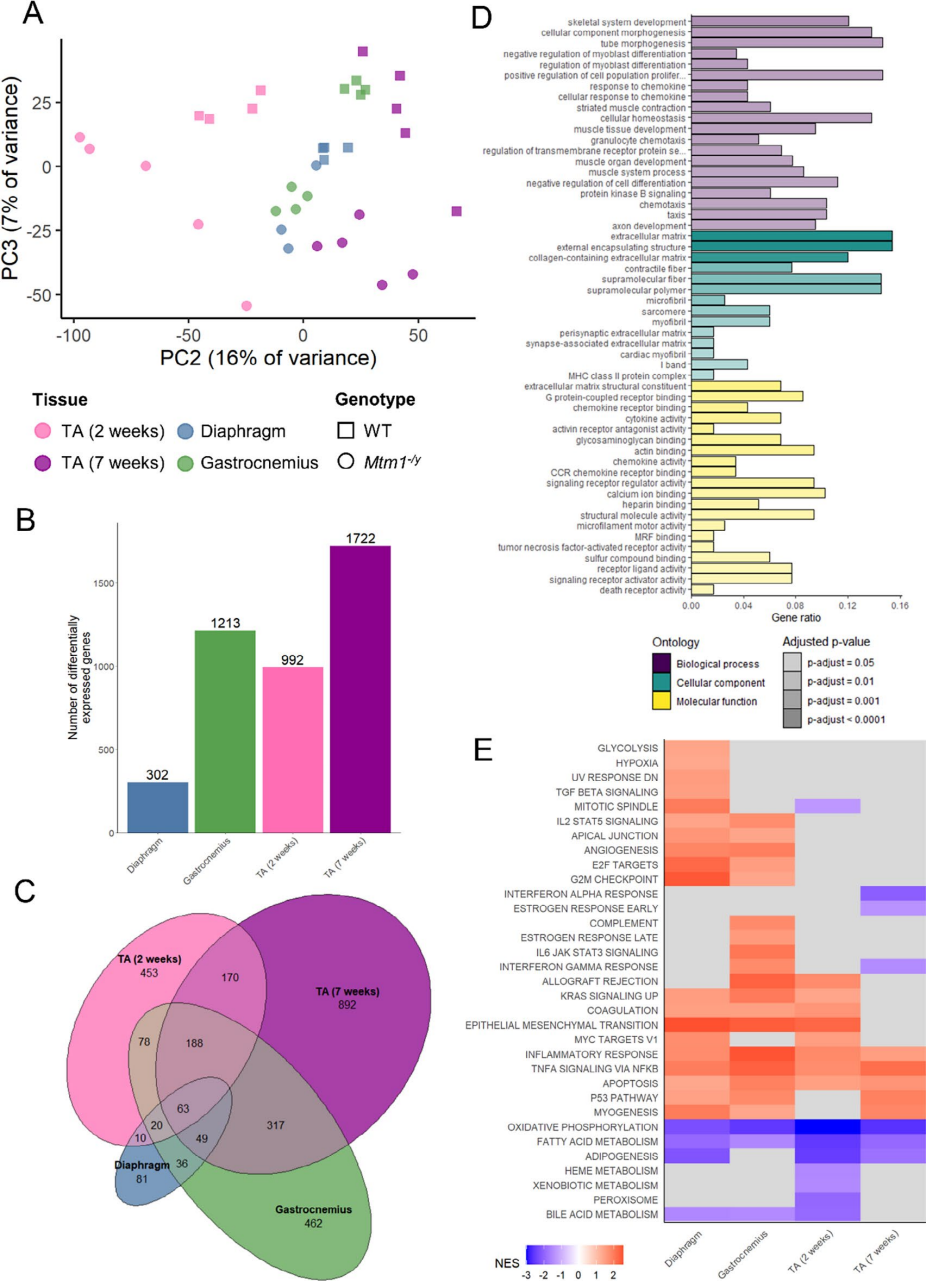
Comparison of 3 skeletal muscles reveals a common disease signature. Exploratory data analysis and differential gene expression analysis of RNA-seq data of several skeletal muscles: diaphragm and gastrocnemius samples (5 weeks) and TA samples (2 and 7 weeks) taken from WT and *Mtm1*^−/y^ mice. **A** Principal component analysis, principal components 2 and 3 are shown. **B** Bar plot showing the number of DEGs between WT and *Mtm1*^−/y^samples for each muscle. **C** Proportional Venn diagram illustrating the number of common and specific DEGs across muscles. **D** Most significant GO terms obtained from over-representation analysis of the 132 DEGs common to all muscles. **E** GSEA results of the “Hallmarks” gene sets obtained from the mouse collection of the Molecular Signature Database in the skeletal muscles. Red and blue respectively indicate statistically significant positive and negative normalized enrichment scores (NES)

## Results

### The effect of XLMTM on gene expression is highly organ-specific

To assess the impact of XLCNM on gene expression in different muscle and non-muscle tissues, we performed RNA-seq in *Mtm1*^−/y^ mice and their wild-type (WT) littermates at 5 weeks, a time point where *Mtm1*^−/y^ mice display muscle weakness without been moribund. Gene expression was quantified in the heart, the liver, and two skeletal muscles: the gastrocnemius and the diaphragm. Exploratory data analysis revealed that clusters of samples derived from gene expression accurately recapitulated organ identity (Fig. 1A-B). Indeed, three clusters which correspond to skeletal muscles, heart, and liver samples were identified. However, the WT and *Mtm1*^−/y^ samples were not clearly separated within each cluster. When considering all organs together, genotype separation only appeared in the gastrocnemius samples.

To evaluate the tissue-specific differences between WT and *Mtm1*^−/y^ mice, we performed differential expression analysis (Fig. 1C). The threshold used to define differentially expressed genes (DEGs) was set at absolute log2-fold change > 1 and adjusted *P* value < 0.05. Gastrocnemius was the tissue with most dysregulation (1213 DEGs), followed by diaphragm (302 DEGs). Gene expression was less affected in heart (68 DEGs) and liver (12 DEGs). Among these dysregulated genes, only two were common to all tissues: *Mtm1* and *Arrdc2*, which were both down-regulated (Fig. 1D, Supplementary Datasets S1-4). The observed downregulation of *Mtm1* in all tissues validated the methodology. The downregulation of *Arrdc2* ranged from a log2FC of -1.25 in the heart to -1.70 in the liver, with a log2FC of -1.45 and − 1.46 in the gastrocnemius and the diaphragm. *Arrdc2* is a mechanosensitive gene, which is downregulated during anabolic stimuli and upregulated during catabolic stimuli in skeletal muscle, its induction leading to thinner myotubes [41, 42]. However, its function in liver and heart is unknown. No dysregulation of *Dnm2* was observed, similarly to previous studies in TA [43]. Apart from these two genes, the liver had no dysregulated genes in common with heart or diaphragm, and only two additional dysregulated genes in common with the gastrocnemius. Similarly, the heart had few dysregulated genes in common with the skeletal muscles (gastrocnemius and diaphragm). On the other hand, skeletal muscles share 168 DEGs, which represents 55% of the dysregulated genes found in diaphragm.

Overall, these data indicate that the effect of XLMTM on the transcriptome is highly organ-specific, both in the number of differentially expressed genes and in the specific genes that undergo alteration. To gain a better understanding of the impact of the disease, we further studied each organ separately.

### The *Mtm1*^-/y^ mouse model shows no liver defects

We investigated whether the *Mtm1*^−/y^ mouse model of XLMTM exhibited hepatic dysfunction similarly to what has been reported in patients. We first studied liver histology following hematoxylin and eosin (H&E) stain. Similar to their WT littermates, the *Mtm1*^−/y^ mice did not exhibit excessive inflammation, fibrosis, steatosis, or necrosis (Fig. 2A). They presented a normal liver architecture into lobules with portal veins, hepatic artery and bile ducts at the periphery, and a central vein at the center. No accumulated bile was visible in the hepatocyte cytoplasm or canaliculi. Immunofluorescent staining of the bile salt export pump (BSEP) showed normal localization in the caniculi, excluding the cholestatic pattern of injury found in patients and *mtm* zebrafish characterized by a quasi-total loss of BSEP expression in the liver [16, 19] (Fig. 2B). Similarly, no liver defects were observed in the histology and BSEP immunofluorescence of 8-month-old *Mtm1*^−/+^ female mice (Supplementary Figure S1).

**Fig. 4:**
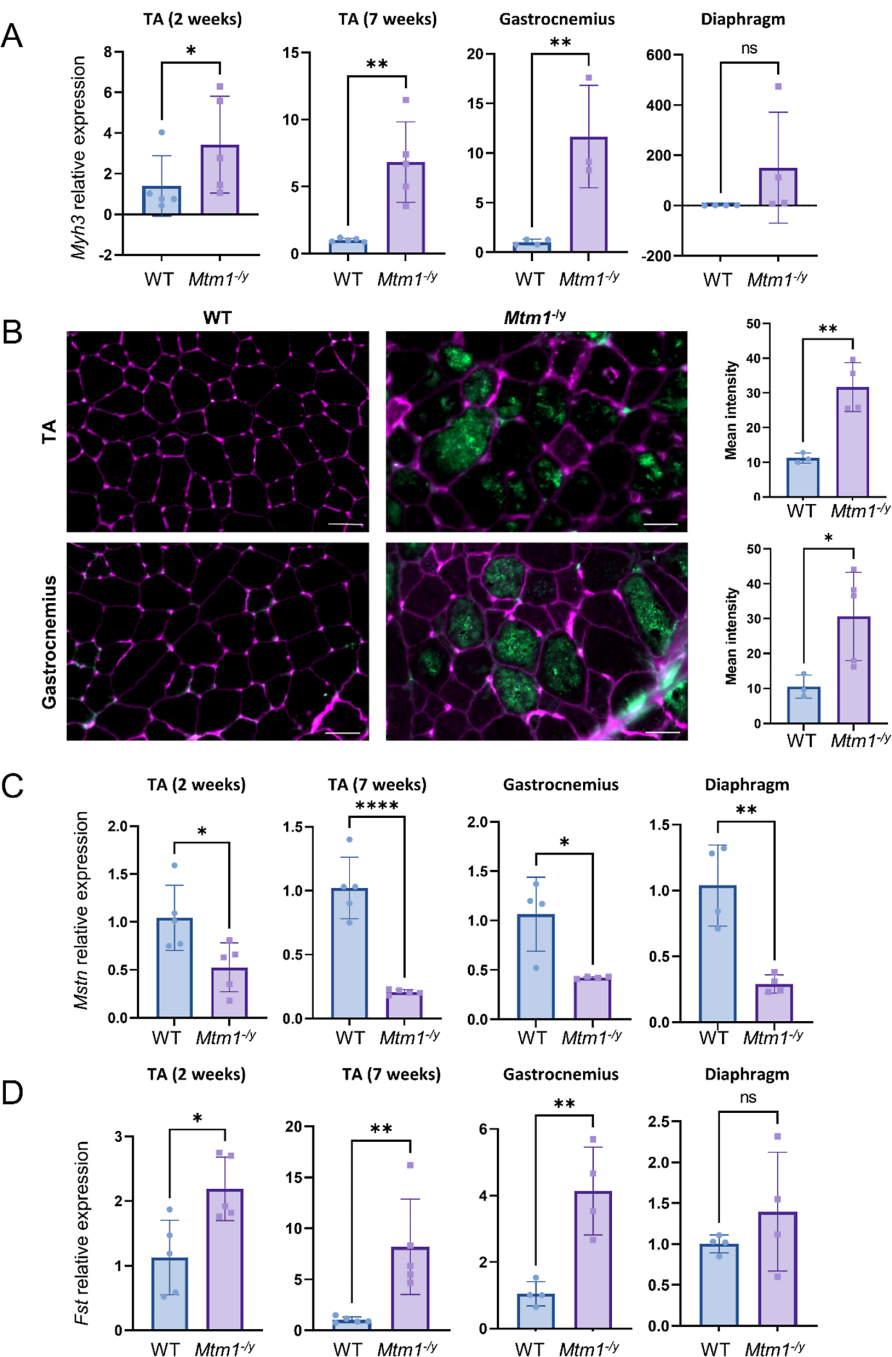
Myogenic development is impaired in the skeletal muscles of *Mtm1*^−/y^mice. **A ***Myh3* transcript relative expression. **B** Representative immunofluorescent staining images of the embryonic myosin (MYH3, green) and WGA (pink) in the tibialis anterior and the gastrocnemius (left), and MYH3 signal intensity quantification (right). Scale bars 50 μm. **C ***Mstn* transcript relative expression. **D ***Fst* transcript relative expression. For panels **A**, **C**, and **D**, transcript expression levels were obtained by RT-qPCR from tibialis anterior (2 weeks and 7 weeks), gastrocnemius and diaphragm (5 weeks) *Mtm1*^−/y^ and WT samples, and are shown as relative expression compared to the average of the WT. ns *P* > 0.05, *P < 0.05, **P < 0.01, ***P < 0.001

As the morphology of the liver of *Mtm1*^−/y^ mice was normal, we proceeded to assess hepatic function by measuring liver enzyme levels in the blood. The levels of bilirubin, aspartate aminotransferase and alanine aminotransferase were within normal ranges in all mice, and no statistically significant differences were found between *Mtm1*^−/y^ and WT mice (Fig. 2C).

Finally, as no defects were detected at the morphological and functional levels, we analyzed RNA-seq data obtained from liver samples. Principal component analysis showed that the first 6 principal components (cumulatively, 99% of variance explained) did not separate the genotypes, which indicated that the WT and *Mtm1*^−/y^ liver samples were highly similar. Indeed, differential gene expression analysis revealed little to no dysregulation in *Mtm1*^−/y^ samples compared to WT. Only 12 differentially expressed genes were identified: *Mtm1*, *Arrdc2*, *Myl2*, *Hhex*, *Myh6*, *Tmc7*, *Jaml*, *Setdb2*, *Pim3*, *Zfp36*, *4632427E13Rik*, and *Phf11c* (Fig. 2D; Supplementary Dataset S1). Due to the low number of DEGs, we then conducted gene set enrichment analysis (GSEA) in order to detect modified biological pathways. Only a small number of gene sets displayed significant enrichment, and none of these were pertinent to hepatic function (Supplementary Figure S2). Quantification of the protein level of DNM2 showed no difference between *Mtm1*^−/y^ and WT livers (Supplementary Figure S3).

Overall, we characterized hepatic morphology, function, and gene expression in *Mtm1*^−/y^ mice, and found no significant differences compared to their WT littermates. This indicates that the unchallenged *Mtm1*^−/y^ mouse model of XLMTM does not replicate the liver defects observed in patients.

### Comparison of 3 skeletal muscles underlines a common disease signature

Unlike the liver, skeletal muscles are severely affected in the *Mtm1*^−/y^ mouse [8, 22, 23] (Supplementary Figure S4). Previous studies have extensively characterized gene expression dysregulation in tibialis anterior [23, 24], a fast—contracting skeletal muscle of the lower leg, but transcriptomic studies of other skeletal muscles are lacking. Therefore, we compared the RNA-seq data we obtained in the gastrocnemius and the diaphragm at 5 weeks with previous RNA-seq data obtained in the tibialis anterior at 2 and 7 weeks [23] to evaluate how MTM1 loss affects different skeletal muscles. Principal component analysis of the cardiac and skeletal muscles samples showed that the tibialis anterior samples clustered properly with the other hindlimb muscle without requiring batch-effect correction (Supplementary Figure S5). By comparing different ages, we aimed to define a fundamental and time-independent common signature.

Principal component analysis revealed a separation according to the different skeletal muscles on the first principal component (45% of variance) and the age on the second PC (16% of variance). Separation of the genotypes appeared when combining the second and third principal components, and was stronger for the tibialis anterior and the gastrocnemius than for the diaphragm (Fig. 3A). Compared to other tissues, the three skeletal muscles showed a high number of DEGs, ranging between 302 and 1722 (Fig. 3B, Supplementary Datasets S3-6). GO term over-representation analysis performed on each muscle identified 238 to 1428 enriched terms with an overlap of 104 terms enriched in all skeletal muscles and related to skeletal muscle development and function (Supplementary Figure S6, Supplementary Datasets S7-10).

In total, there were 132 common DEGs affected in the three studied skeletal muscles at any age (Fig. 3C, Supplementary Dataset S11). Enrichment analysis revealed that these genes were associated to GO terms previously identified in the tibialis anterior such as skeletal muscle development and function, extracellular matrix, and inflammation, but also revealed new affected pathways: calcium homeostasis and protein kinase B (AKT1) signaling, and negative regulation of cell development (Fig. 3D, Supplementary Dataset S12). Investigation of the cell development pathways revealed an upregulation of the fetal myosin *Myh3*, which was confirmed by RT-qPCR and immunofluorescence microscopy (Fig. 4A-B). Moreover, the upregulation of *Fst* and downregulation of *Mstn* were identified on the RNA-seq data and confirmed by RT-qPCR (Fig. 4C-D).

**Fig. 5:**
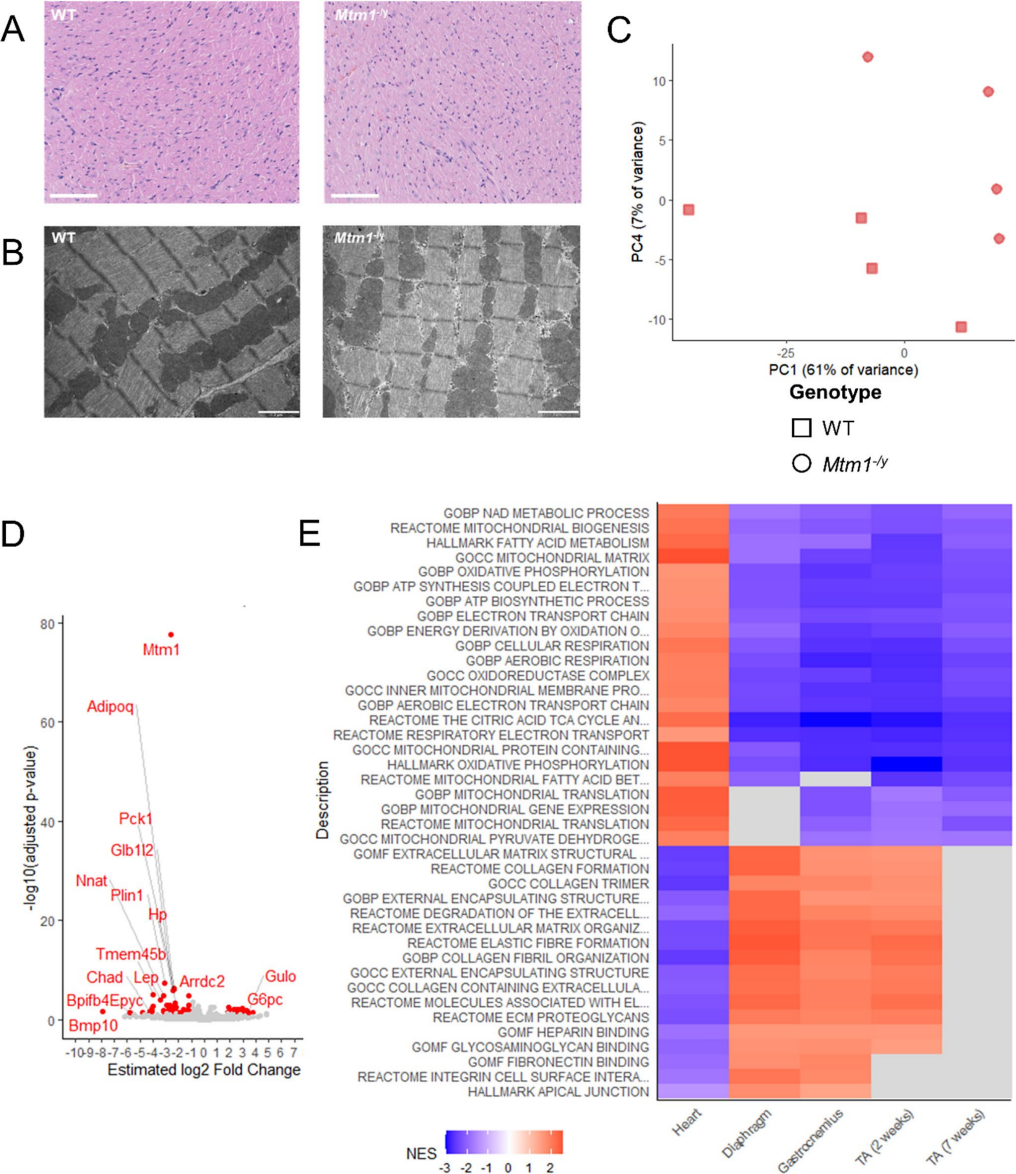
The *Mtm1*^−/y^ mouse heart shows no structural defects but is affected at the transcriptomic level. **A** Representative heart histology of WT and *Mtm1*^−/y^ mice (H&E). Scale bars 100 μm. **B** Representative heart electron microscopy of WT and *Mtm1*^−/y^ mice (H&E). Scale bars 2 μm. **C** PCA plot of heart samples, PC1 and PC4 are shown. **D** Volcano plot showing the 68 DEGs in heart. **E** Subset of the GSEA results based on Molecular Signature Database Hallmarks, Reactome pathways, and GO terms gene sets in cardiac and skeletal muscles. Red and blue respectively indicate statistically significant positive and negative normalized enrichment scores (NES)

To evaluate the robustness of this common disease signature, we compared it to the list of differentially expressed genes found in XLMTM dogs [44]. Out of 132 common DEGs, 25 were also differentially expressed in the vastus lateralis or biceps femoris of the canine model of XLMTM, including *Mstn*, *Cilp*, and *Tiam2* (Supplementary Dataset S13).

To investigate further the functional consequences of gene dysregulation caused by XLMTM in skeletal muscles, we performed gene set enrichment analysis (GSEA) on a list of 50 curated gene sets representing well-defined biological states and processes and obtained from the Molecular Signature Database (MSigDB). From these gene sets, we were able to identify pathways that were significantly affected in all skeletal muscles at all time points (Fig. 3E). In particular, the inflammatory response, the TNFα signaling via NFκB, and the apoptosis gene sets were positively enriched, while the fatty acid metabolism and oxidative phosphorylation gene sets had a significant negative enrichment score when comparing to WT. We then performed GSEA on 1251 gene sets obtained from the Reactome database to acquire a more refined understanding of the impacted pathways. Over 10 pathways related to mitochondrial function were depleted in at least 3 groups, which reinforced the hypothesis that oxidative phosphorylation is impaired in skeletal muscles. Additionally, several pathways related to extracellular matrix organization were enriched in the gastrocnemius, the diaphragm, and the tibialis anterior at 2 weeks (Supplementary Figure S7).

To evaluate whether these results were consistent with other transcriptomic studies in mice, independently of the genetic background and breeding/sequencing conditions, we compared them to TA and quadriceps datasets previously published [24, 25]. After removal of batch effects with the limma package, principal component analysis revealed that PC3 (11% of variance) separated the ages while genotype separation occurred with PC4 (6% of variance), regardless of the skeletal muscle or genetic background (Supplementary Figure S8A-B). GSEA revealed common dysregulated pathways in symptomatic muscles, such as oxidative phosphorylation, TNFα signaling via NFκB and epithelial mesenchymal transition, which were in agreement with the results of our study (Supplementary Figure S8C).

Overall, these results identified a disease signature common to several skeletal muscles. By considering only these 132 DEGs that were dysregulated in all muscles, we were able to confirm previous results obtained in the tibialis anterior and underline new pathways of interest.

### The *Mtm1*^-/y^ mouse heart shows a mild phenotype

Myopathies can be associated to cardiac phenotypes, and some genes are implicated in both myopathy and cardiomyopathy, such as *TTN* and *SPEG* [45–47]. Even though skeletal muscles are severely affected in XLMTM, the cardiac function of *Mtm1*^−/y^ mice has never been extensively studied. We thus performed functional phenotyping, histological and ultrastructural analyses, and gene expression quantification in *Mtm1*^−/y^ mice.

First, we used noninvasive blood pressure measurement, electrocardiography (ECG), and echography to obtain a functional phenotyping of the heart at 5–6 weeks. No significant changes in heart rate and systolic blood pressure were observed in *Mtm1*^−/y^ mice compared to WT (Table 2). On the ECG, a mild increase in ST segment elevation was observed in the *Mtm1*^−/y^ mice, but no statistically significant differences were detected for the RR, PR, QRS, ST, and QT intervals durations, nor for the P-wave duration and R wave-amplitude (Table 1). Finally, heart echography revealed mild decreases in aortic artery diameter, stroke volume and tricuspid valve velocity time integral, but no statistically significant differences in all other measured parameters (Table 3).

**Table 2:** Electrocardiographic data

	WT	Mtm1^−/y^	*P* value	Significance
RR interval (ms)	161.8 ± 7.5	172.9 ± 8.1	0.33	ns
PR interval (ms)	48.9 ± 0.9	48.2 ± 0.8	0.56	ns
P-wave duration (ms)	22.3 ± 1.6	24.0 ± 0.7	0.36	ns
QRS interval (ms)	19.4 ± 3.2	16.4 ± 0.6	0.37	ns
R-wave amplitude (mV)	1.20 ± 0.14	1.58 ± 0.11	0.06	ns
ST segment elevation (mV)	0.27 ± 0.06	0.41 ± 0.01	0.03	*
ST interval (ms)	49.7 ± 6.0	47.1 ± 1.1	0.67	ns
QT interval (ms)	63.8 ± 6.8	59.4 ± 1.4	0.53	ns

Values are mean ± SEM. *n* = 8 mice in each group. *: *P*-value < 0.05, ns: not statistically significant (*P* value > 0.05)

**Table 3:** Echocardiographic data

	WT	Mtm1^−/y^	*P* value	Significance
LVID d (mm)	3.86 ± 0.20	3.60 ± 0.08	0.25	ns
LVID s (mm)	2.69 ± 0.24	2.53 ± 0.13	0.56	ns
LV Vol d (µL)	66.3 ± 7.3	54.7 ± 3.1	0.16	ns
LV Vol s (µL)	29.5 ± 5.6	23.7 ± 3.2	0.39	ns
LVPW d (mm)	0.70 ± 0.04	0.68 ± 0.03	0.61	ns
LVPW s (mm)	0.96 ± 0.07	0.91 ± 0.02	0.52	ns
LVAW d (mm)	0.68 ± 0.01	0.68 ± 0.03	0.94	ns
LVAW s (mm)	0.99 ± 0.05	0.95 ± 0.04	0.52	ns
EF (%)	57.0 ± 5.3	57.6 ± 3.5	0.93	ns
FS (%)	30.6 ± 4.3	29.9 ± 2.2	0.89	ns
LV mass (AW) (g)	92.0 ± 7.1	80.3 ± 4.9	0.19	ns
AET (ms)	54.0 ± 1.5	55.8 ± 1.2	0.35	ns
IVCT (ms)	19.3 ± 1.5	17.1 ± 1.6	0.34	ns
IVRT (ms)	23.2 ± 1.5	23.3 ± 2.2	0.97	ns
MV E (mm/s)	653 ± 54	608 ± 29	0.48	ns
MV A (mm/s)	480 ± 49	460 ± 41	0.76	ns
MV E/A	1.39 ± 0.07	1.36 ± 0.07	0.33	ns
AoV diameter (mm)	1.48 ± 0.01	1.41 ± 0.02	0.007	**
AoV VTI (mm)	24.5 ± 1.3	20.2 ± 2.9	0.16	ns
AoV SV (µL)	42.2 ± 2.3	32.61 ± 3.0	0.02	*
AoV CO (mL/min)	15.9 ± 1.2	12.9 ± 1.5	0.14	ns
TV VTI (mm)	19.25 ± 0.8	15.8 ± 1.0	0.02	*
TV E	342 ± 52	279 ± 38	0.35	ns
TV A	460 ± 39	367 ± 21	0.06	ns
TV E/A	0.76 ± 0.12	0.81 ± 0.16	0.15	ns

Values are mean ± SEM. *n* = 8 mice in each group. LVID d: Left ventricular end-diastolic diameter; LVID s: Left ventricular end-systolic diameter; LV Vol d: Left ventricular end-diastolic volume; LV Vol s: Left ventricular end-systolic volume; LVPW d: Left ventricular end-diastolic posterior wall thickness; LVPW s: Left ventricular end-systolic posterior wall thickness; LVAW d: Left ventricular end-diastolic anterior wall thickness; LVAW s: Left ventricular end-systolic anterior wall thickness; EF: Ejection fraction; FS: Fractional shortening; LV: Left ventricular; AW: Anterior wall thickness; AET: Aortic ejection time; IVCT: Isovolumic contraction time; IVRT: Isovolumic relaxation time; MV: Mitral valve; E: Early ventricular filling velocity; A: Late ventricular filling velocity; AoV: Aortic valve; VTI: Velocity time integral; SV: Stroke volume; CO: cardiac output; TV: tricuspid valve. *: *P*-value < 0.05, **: *P*-value < 0.01, ns: not statistically significant (*P* value > 0.05)

We thus investigated further heart morphology and ultrastructure. Heart slices were stained with hematoxylin and eosin (Fig. 5A). Compared to their WT littermates, the *Mtm1*^−/y^ mice did not exhibit any striking abnormalities in terms of inflammation, necrosis, fibrosis, or cardiomyocytes hypertrophy. To study subcellular structure and organization, we then conducted transmission electron microscopy. This demonstrated a normal organization of sarcomere, contractile filaments, mitochondria and membrane structures (Fig. 5B).

Overall, while these data suggested the presence of a mild cardiac phenotype without obvious structural alterations once the myopathy is well established. Compared to the skeletal muscle defects that include strong muscle weakness, muscle and myofiber hypotrophy, and disorganization of sarcomeres and organelles [48] (Supplementary Figure S4), the cardiac phenotype is very mild.

To investigate the underlying reasons for the substantial difference in severity between cardiac (minor defects) and skeletal muscle (strong impairment and disorganization), we analyzed RNA-seq data obtained from cardiac muscle samples of *Mtm1*^−/y^ and WT mice. Principal component analysis showed that PC1 and PC4 (68% cumulative variance) separated the genotypes (Fig. 5C). Differential gene expression analysis revealed 68 DEGs (Fig. 5D), and GSEA revealed a 664 significantly affected pathways (Fig. 5E).

### Cardiac and skeletal muscles display an opposite dysregulation pattern at the transcriptome level

Strikingly, several affected pathways in the heart were inversely affected in the skeletal muscles (Fig. 5E; Supplementary Figure S9, Supplementary Dataset S14). For example, the common skeletal muscle signature identified in this study included upregulation of pathways related to the extracellular matrix and cell adhesion, and downregulation of pathways related to mitochondrial function (oxidative phosphorylation, complex I biogenesis, respiratory electron transport, fatty acid metabolism…), and the dysregulation of these pathways was inverted in the heart.

**Fig. 6:**
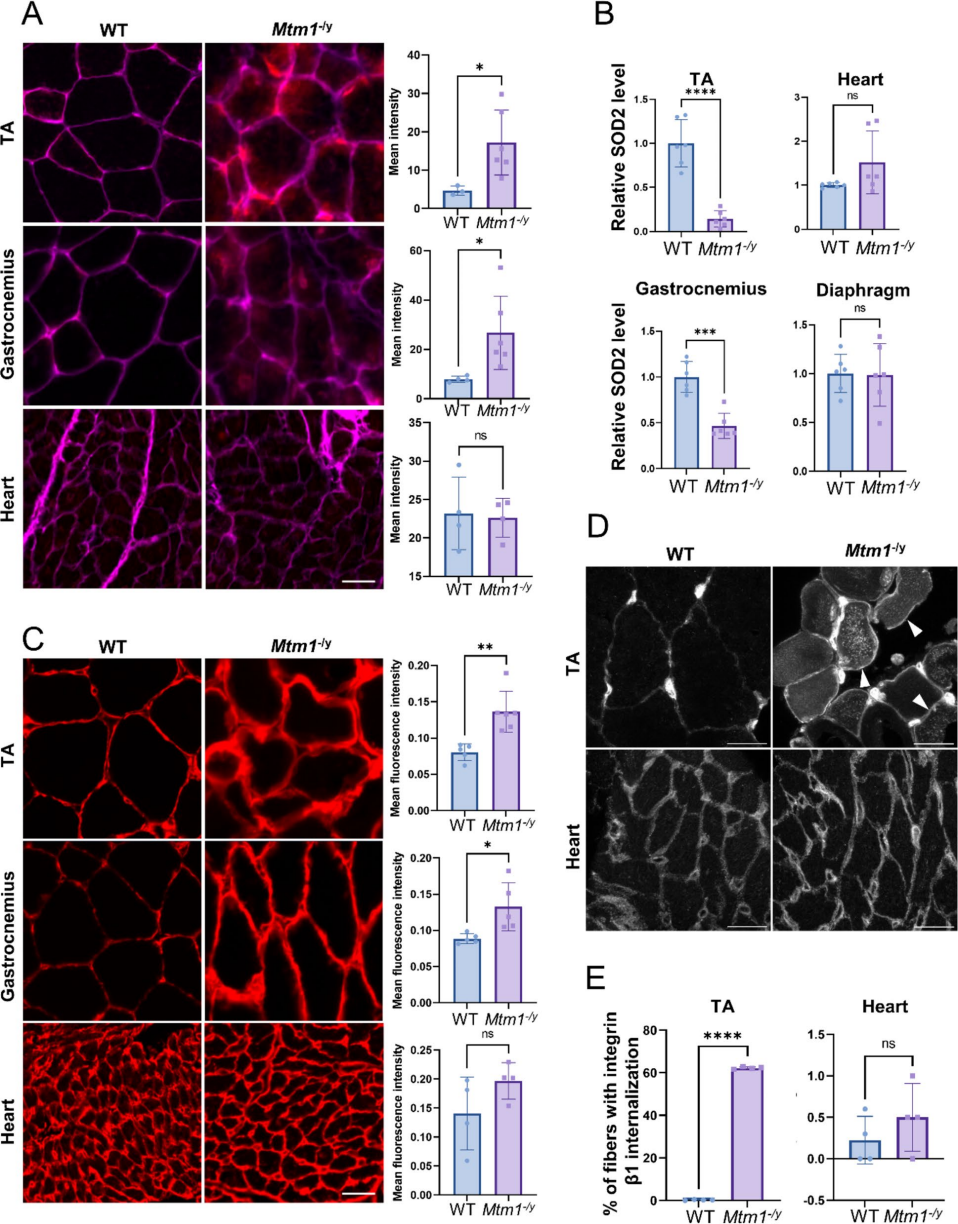
Investigation of the pathways inversely dysregulated in the heart compared to the skeletal muscles. **A** ROS quantification with DHE assay in tibialis anterior (TA), gastrocnemius and heart. Representative fluorescent images (left) and ROS signal intensity quantification (right). Same scale for all images, scale bar 20 μm. **B** Protein level of SOD2 in TA, diaphragm, gastrocnemius and heart of WT and *Mtm1*^−/y^ mice (*n* = 6) obtained by western blotting with standardization by Ponceau red staining. The fold difference from the average of the WT is shown. **C** Representative immunofluorescent staining images of laminin (left) and signal intensity quantification in a 2 μm-wide pericellular region (right) in tibialis anterior (TA), gastrocnemius and heart. Same scale for all images, scale bar 20 μm. **D** Representative immunofluorescent staining images of integrin β1 in TA and heart. Arrows indicate examples of fibers showing abnormal localization of integrin β1. Scale bars 20 μm. **E** Quantification of fibers with abnormal integrin β1 localization in TA and heart (*n* = 4). *Mtm1*^−/y^ and WT littermates samples were taken at 7 weeks for all panels. Student’s t-test: ns *P* > 0.05, *P < 0.05, **P < 0.01, ***P < 0.001

Impaired mitochondrial function, in particular oxidative phosphorylation deficiency, is associated with an increased production of reactive oxygen species (ROS) and an increase in the level of the ROS detoxification enzyme SOD2, a superoxide dismutase located in the mitochondria [49]. Thus, we quantified ROS in skeletal and cardiac muscle with a dihydroethidium (DHE) assay (Fig. 6A), which showed elevated levels of ROS in TA and gastrocnemius but no difference in the heart. Then, we measured the levels SOD2 to evaluate whether antioxidant defenses against ROS varied in skeletal versus cardiac muscles. SOD2 was downregulated in tibialis anterior and gastrocnemius with no statistical differences in heart and diaphragm (Fig. 6B, Supplementary Figure S10A-D).

Extracellular matrix (ECM) defects are well known in skeletal muscle affected by XLMTM, with smaller and rounder myofibers and increased inter-fiber space [3]. Therefore, we monitored laminin in cardiac and skeletal muscle with immunofluorescent assays, which revealed a thicker laminin signal around skeletal muscle fibers but not in the heart (Fig. 6C). Additionally, MTM1 has been shown to control focal adhesion and integrins recycling [3, 4]. We performed immunofluorescence staining to quantify the amount of cardiac and skeletal muscle fibers presenting abnormal β1 integrin localization. We confirmed abnormal β1 integrin internalization in tibialis anterior of *Mtm1*^−/y^ mice, while no defects were detected in the heart (Fig. 6D-E).

**Fig. 7:**
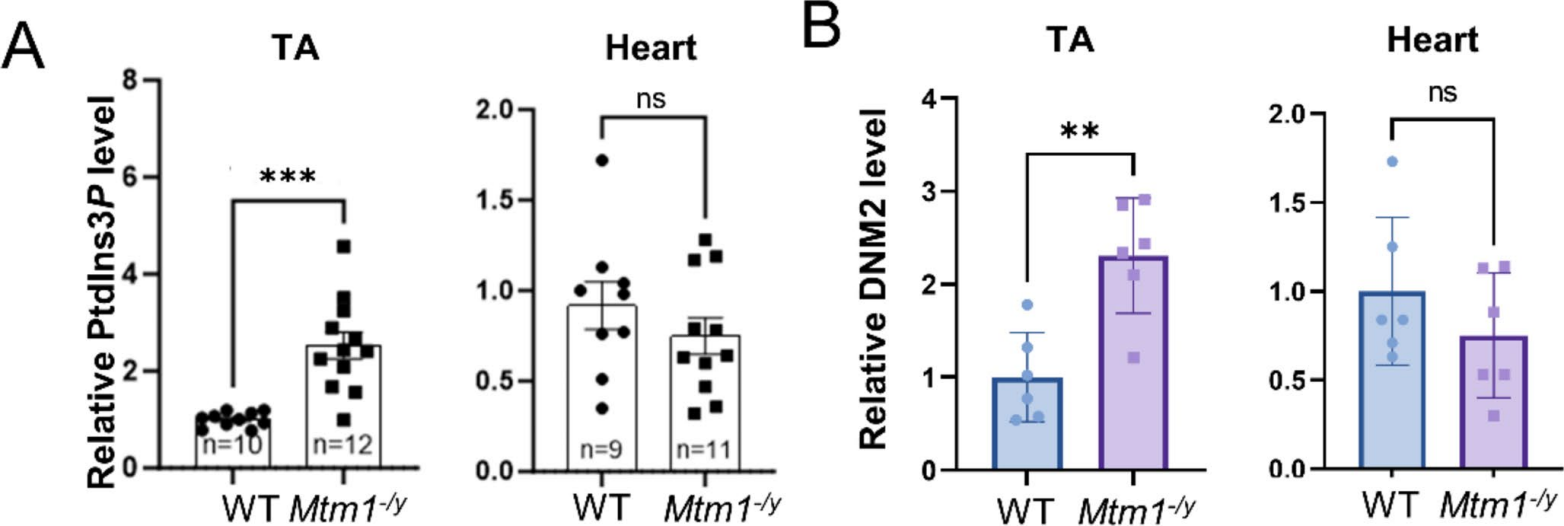
Investigation of PtdIns3*P* and DNM2 levels in the heart and TA. **A** PtdIns3*P* level in *Mtm1*^−/y^ TA and heart as the fold difference from the average of the WT at 7 weeks. **B** Protein level of DNM2 in tibialis anterior (TA) and heart of WT and *Mtm1*^−/y^ mice (*n* = 6) obtained by western blotting with standardization by Ponceau red staining in *Mtm1*^*−/y*^ and WT mice at 7 weeks, represented as the fold difference from the average of the WT. Student’s t-test: *P < 0.05, **P < 0.01, ***P < 0.001

The main substrate of MTM1 is PtdIns3*P*. PtdIns3*P* is composed of two fatty acid chains and one inositol molecule phosphorylated at position 3 of the ring [50]. In addition, PtdIns3*P* and MTM1 are main regulators of protein endocytosis and recycling [51], including integrins [52]. Therefore, we measured PtdIns3*P* levels in WT and *Mtm1*^−/y^ cardiac and tibialis anterior muscles. While the level of PtdIns3*P* was significantly increased in *Mtm1*^−/y^ tibialis anterior as expected, we observed no significant dysregulation in the heart (Fig. 7A). In addition, we assessed the level of DNM2, another protein mutated in CNM and a key regulator of membrane and protein trafficking. Western blot analysis revealed a 2.2-fold increase of DNM2 in tibialis anterior muscle and a normal level in heart in *Mtm1*^−/y^ mice compared to WT mice (Fig. 7B; Supplementary Figure S10E-F).

In conclusion, we found an inverted dysregulation of specific pathways in heart compared to skeletal muscle at the transcriptome level. We confirmed the strong alteration of these pathways in skeletal muscle at the biochemical level while they were unaffected in heart. These data, combined to the good preservation of heart function and morphology, suggest that the modulation of these pathways constitutes a molecular compensatory mechanism in heart.

## Discussion

In this study, we investigated potential organ-specific defects linked to XLMTM in the *Mtm1*^*−/y*^ mouse model. RNA-seq investigations of several skeletal muscles, heart, and liver highlighted that transcriptional alterations are highly organ-specific. A limitation of this present study is the comparison of transcriptome from different muscles and ages, nevertheless comparison of our data obtained on different muscles and with previously published datasets highlighted similar pathways in the different muscle types and ages. A disease signature common to several skeletal muscles including diaphragm confirmed dysregulation of muscle development, inflammation, cell adhesion and extracellular matrix, and revealed novel altered pathways such as oxidative phosphorylation. Conversely to skeletal muscles which are affected by severe muscle weakness and major myofiber disorganization and hypotrophy, heart displayed only mild functional alterations without obvious structural defects. Concordantly, we found an inverted dysregulation of specific pathways in heart compared to skeletal muscle at the transcriptome level. Indeed, biochemical and cellular experiments showed that mitochondrial function as well as cell adhesion and beta integrin trafficking were strongly affected in skeletal muscle and normal in cardiac muscle. Moreover, biomarkers reflecting the molecular activity of MTM1, as PtdIns3*P* and DNM2 levels, were significantly increased in the skeletal muscle of the *Mtm1*^*−/y*^ mouse but not in cardiac muscle, suggesting a compensatory mechanism in heart. Concerning liver function and morphology, there were no defects noted in the *Mtm1*^*−/y*^ mouse.

### Hepatic function in XLMTM

XLMTM male patients may display hypertransaminemia, hyperbilirubinemia and intrahepatic cholestasis [16–18], and these signs were exacerbated with AAV-MTM1 gene therapy. In addition, long-term survivors sometimes die from hepatic peliosis. Functional biochemistry, histology and transcriptomic analyses did not reveal any liver defects *Mtm1*^*−/y*^ mouse when they display a clear skeletal muscle phenotype. While it would be interesting to investigate liver integrity at older ages, the *Mtm1*^*−/y*^ mice die from the myopathy at about 5–9 weeks. Female *Mtm1*^−/+^ mice do not present obvious motor defects and live longer than the *Mtm1*^−/y^ males. Our study shows that heterozygous deficiency of *Mtm1* is not sufficient to cause detectable liver defects in these mice. A possibility would be to characterize liver function in the milder *Mtm1*^*R69C/y*^ mouse engineered with a missense mutation linked to a mild patient phenotype [53]. However, preliminary analysis of this model showed normal liver with no sign of hepatic peliosis. It is possible this mutation may associate the mild myopathy with a weaker impact on liver. Another option would be to challenge the *Mtm1*^−/y^ mouse models in order to induce liver defects. Nevertheless, it is still unclear if there is a trigger for this phenotype in patients, and what it could be, although vaccination or infection preceded liver defects in several cases [17]. Lastly, the MTM1 function in liver might vary between mice and humans. As a result, the mouse might not serve as the ideal animal model to study this particular phenotype. Of note, liver histology was found normal in XLMTM dogs with an MTM1 mutation, while *mtm* zebrafish displayed hepatic cholestasis with impaired bile flux and structural abnormalities of the bile canaliculus [19, 54].

### Potential compensatory mechanisms in cardiac versus skeletal muscles

Through a comparative transcriptomic analysis of tibialis anterior, gastrocnemius and diaphragm, we identified dysregulated pathways common to different skeletal muscles, and thus probably representing the main causes and consequences of XLMTM. It confirmed dysregulation of muscle development and contraction, cell adhesion and extracellular matrix, mitochondrial metabolism, and inflammation [23, 24, 44]. Here, we also validated a decreased expression of SOD2, a mitochondrial superoxide dismutase regulating ROS, and the abnormal retention of intracellular of β1 integrin. These findings helped in deciphering the pathomechanism of the disease. Defects in muscle development and contraction could underly the early onset and severe muscle weakness, and cell adhesion alterations would lead to the smaller and rounder myofibers with centralized nuclei that are the histopathological hallmarks of XLMTM [6–8].

Conversely to skeletal muscles, only a mild cardiac phenotype without obvious structural alterations was detected. It is envisaged that the few altered cardiac parameters in the *Mtm1*^*−/y*^ mouse are related to the pronounced skeletal muscle and respiratory failures. Indeed, the vast majority of XLMTM patients die from respiratory failure. In two studies covering 145 and 50 XLMTM patients, a single case of cardiomyopathy was reported [55, 56].

The extreme difference in severity between cardiac and skeletal muscles suggests a compensatory mechanism preserving the heart that is not present in skeletal muscle. It was thus important to investigate the heart transcriptome as it could highlight key pathomechanisms and potential therapeutic targets. We found an inverted dysregulation of specific pathways in heart compared to skeletal muscle at the transcriptome level in the *Mtm1*^*−/y*^ mouse. Specifically, cell adhesion and integrins pathways were upregulated in muscle and downregulated in heart. Conversely, mitochondrial function and metabolism pathways were downregulated in skeletal muscle and upregulated in heart. We confirmed through biochemical and immunolocalization experiments of SOD2 and β1 integrin that these pathways are indeed altered in skeletal muscle but normal in heart. Therefore, in *Mtm1*^−/y^ mice, the liver is unaffected at all levels, but the heart is sensitive to myotubularin deficiency, as shown by the separation the genotypes on the PCA (Fig. 5C), and the dysregulation of pathways at the transcriptomic level identified with GSEA (Fig. 5D). Therefore, several of these pathways may act as a compensatory mechanism and prevent the cellular and functional phenotypes observed in skeletal muscles. These patterns of opposite dysregulation highlight that these pathways represent key pathomechanism leading to skeletal muscle disorganization and weakness.

Previous work has highlighted similar compensatory mechanisms in dysferlin-deficient muscular dystrophy, a muscular disorder without myocardial involvement. Using microarray gene expression profiling, the authors showed an upregulation of CD55, a complement inhibitory factor, in the heart compared to a downregulation in skeletal muscle. Loss of CD55 leads to an increased susceptibility to complement attack in skeletal muscle, and its upregulation in the heart may explain the preserved cardiac function [57]. Subsequent work showed that genetic disruption of the complement improved the muscle phenotype in dysferlin-deficient mice [58], which shows that the pathways implicated in compensatory mechanisms in non-affected cardiac muscle can be promising therapeutic targets to cure the disease in affected skeletal muscles.

To investigate further the mechanism of this opposite dysregulation, we investigated specific molecules linked to MTM1 function and CNM. The main substrate of MTM1, PtdIns3*P*, was increased in skeletal muscle and normal in heart in the *Mtm1*^*−/y*^ mouse. Such compensation in heart might be due to overactivation of MTM1 homologs sharing the similar enzymatic activity. However, no MTM-related (MTMRs) genes were found differentially expressed in our transcriptome data. Such overactivation may be happening at the protein level. Of note, exogenous expression of MTMR2 in the muscle of the *Mtm1*^*−/y*^ mouse rescued the phenotypes [23, 43, 48, 59, 60]. Also, normalizing PtdIns3*P* level through inhibition of the PI3-kinase PIK3C2B rescued the *Mtm1*^*−/y*^ mouse [61, 62]. Another molecular explanation of the preserved function observed in heart may be DNM2, also mutated in CNM. Indeed, DNM2 protein level is increased in skeletal muscle of the *Mtm1*^*−/y*^ mouse, an induced increase of DNM2 in WT mice created a myotubular myopathy phenotypes, and normalization of DNM2 level fully rescued the *Mtm1*^*−/y*^ mouse [31, 43, 48]. Interestingly, DNM2 level is not altered in the heart of *Mtm1*^*−/y*^ mouse. A third molecule which may underly the preserved function in heart is BIN1. BIN1 is also mutated in CNM, exogenous expression of BIN1 efficiently rescued the *Mtm1*^*−/y*^ mouse phenotypes including integrin trafficking [3], and different splice isoforms of BIN1 are expressed in cardiac and skeletal muscle [63]. Exon 11 and 13 are respectively specific from skeletal or cardiac muscles.

Overall, by performing a multi-organ transcriptomic study in *Mtm1*^−/y^ mice, we found patterns of dysregulation suggesting compensatory mechanisms in the heart, an organ that is not affected in XLMTM. These pathways represent exciting therapeutic targets to cure the skeletal muscle phenotype caused by XLMTM.

## Electronic supplementary material

Below is the link to the electronic supplementary material.

Supplementary Material 1

Supplementary Material 2

## Data Availability

RNA-sequencing data for heart, liver, diaphragm and gastrocnemius samples have been deposited in NCBI’s Gene Expression Omnibus and are accessible through GEO Series accession number GSE242761. RNA-sequencing data for tibialis anterior was obtained from a previous study and is accessible through GEO Series accession number GSE160084 [23]. The R code and data necessary to reproduce the bioinformatics analysis are available on Zenodo: 10.5281/zenodo.14184555. All other data underlying the results are available in the article and its supplemental material.

## Abbreviations

AAV: Adeno-associated virus
CNM: Centronuclear myopathies
DEGs: Differentially expressed genes
ECG: Electrocardiography
GSEA: Gene set enrichment analysis
PtdIns3*P*: Phosphatidylinositol 3-phosphate
TA: Tibialis anterior
XLMTM: X-Linked myotubular myopathy
