# Supplementary material for: Potential compensatory mechanisms preserving cardiac function in myotubular myopathy
Source: Cell Mol Life Sci. 2024 Dec 3;81(1):476. doi: 10.1007/s00018-024-05512-9 (PMC11615164; doi:10.1007/s00018-024-05512-9)
Supplement: Supplementary file 2 — Supplementary Material 2 [file 18_2024_5512_MOESM2_ESM.pdf]

Supplementary figures

Figure S1. Liver structure of Mtm1-/+ females at 8 months.

Figure S2. Gene set enrichment analysis results of MSigDb "Hallmarks" gene sets in the liver

Figure S3. DNM2 Western Blot in the liver of *Mtm1*$^{-/y}$ and WT mice

Figure S4. Skeletal muscle defects in *Mtm1*$^{-/y}$ mice compared to WT

Figure S5. Principal component analysis of heart, gastrocnemius, diaphragm and tibialis anterior samples

Figure S6. Venn diagram of enriched GO terms in gastrocnemius, diaphragm, tibialis anterior (2 weeks and 7 weeks)

Figure S7. Gene set enrichment analysis results of Reactome pathways gene sets in the skeletal muscles

Figure S8. Comparison of RNA-seq datasets obtained from skeletal muscles of 129Pas and C57BL/6J *Mtm1*$^{-/y}$ and WT mice

Figure S9. Gene set enrichment analysis results of MSigDb Hallmarks, Reactome pathways and GO terms gene sets in cardiac and skeletal muscles.

Figure S10. SOD2 and DNM2 Western Blots.

Figure S1

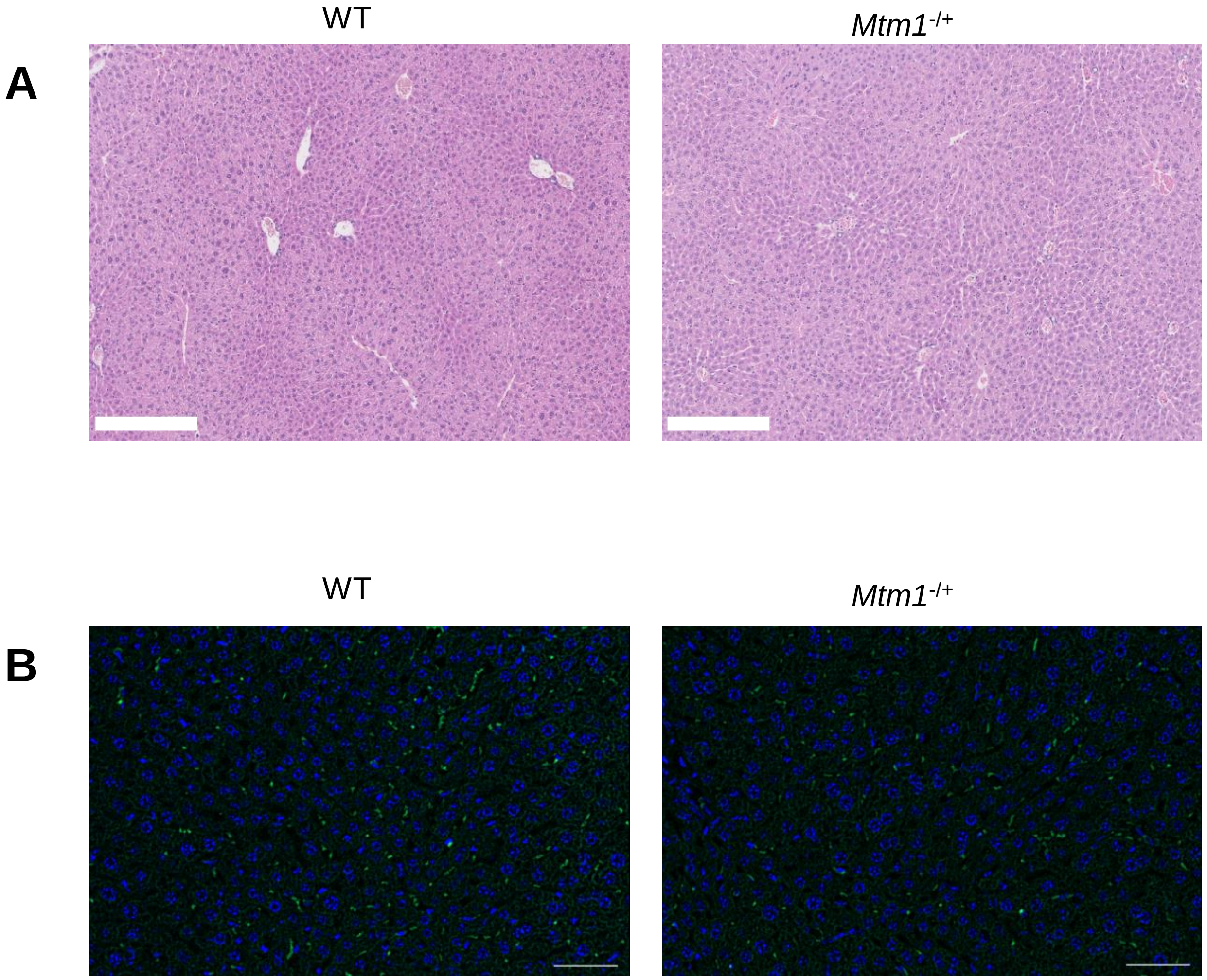

Figure S1. **Liver structure of *Mtm1*$^{-/+}$ females at 8 months. (A)** Hematoxylin and eosin histology (scale bar 250µm). (**B)** BSEP immunofluorescence (same scale bar for both images: 50µm)

Figure S2

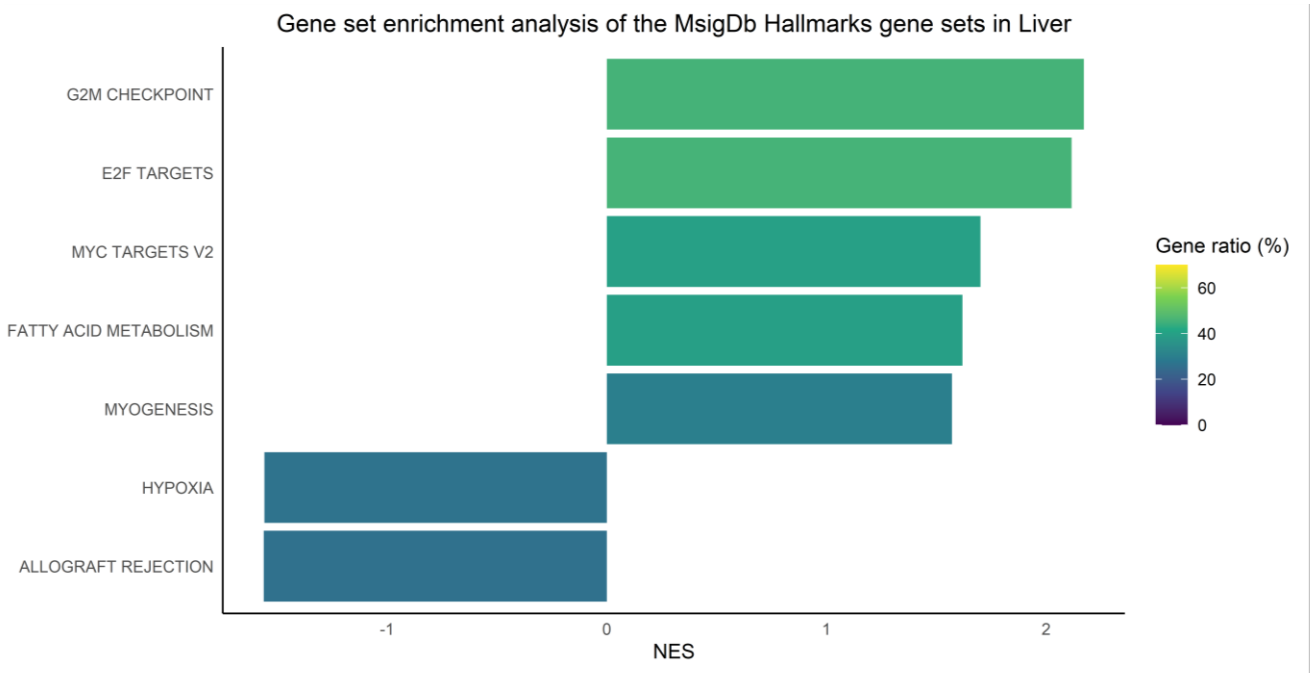

Figure S2. **Gene set enrichment analysis results of MSigDb "Hallmarks" gene sets in the liver.** Only statistically significant enrichment results are shown (BH adjusted *P* value < 0.05). Gene sets were taken from the "Hallmarks" mouse collection in the Molecular Signature Database (MsigDb). The gene ratio is computed as the number of leading edge genes divided by the total number of genes in the gene set. NES: Normalized Enrichment Score

Figure S3

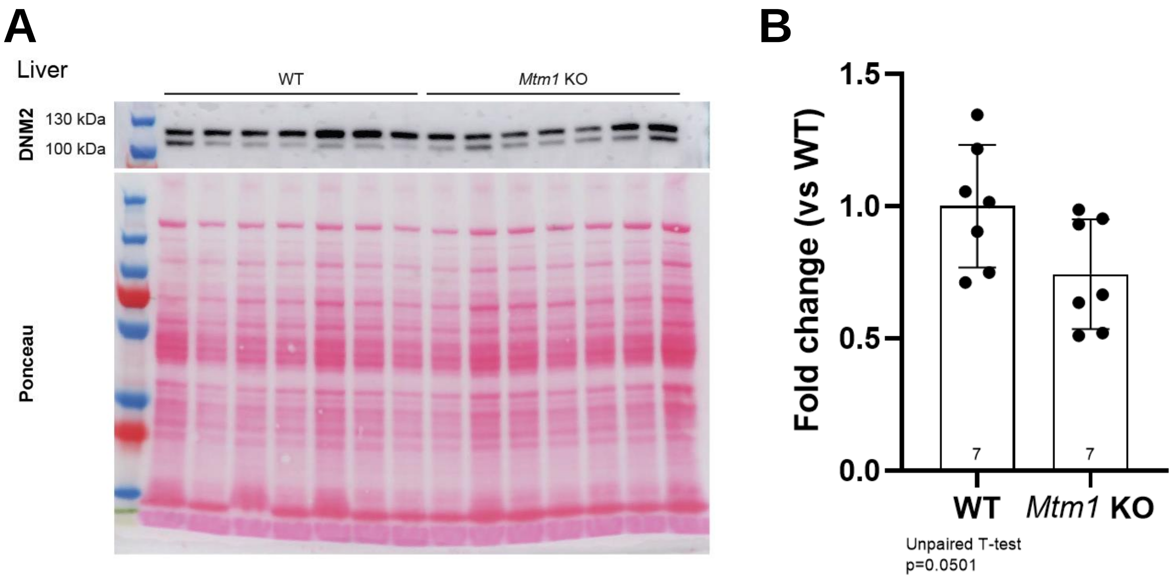

Figure S3. **DNM2 Western Blot in the liver of *Mtm1*$^{-/y}$ and WT mice. (A)** Immunostained DNM2 bands (top), Ponceau-stained full blot (bottom) **(B)** Relative DNM2 expression level

Figure S4

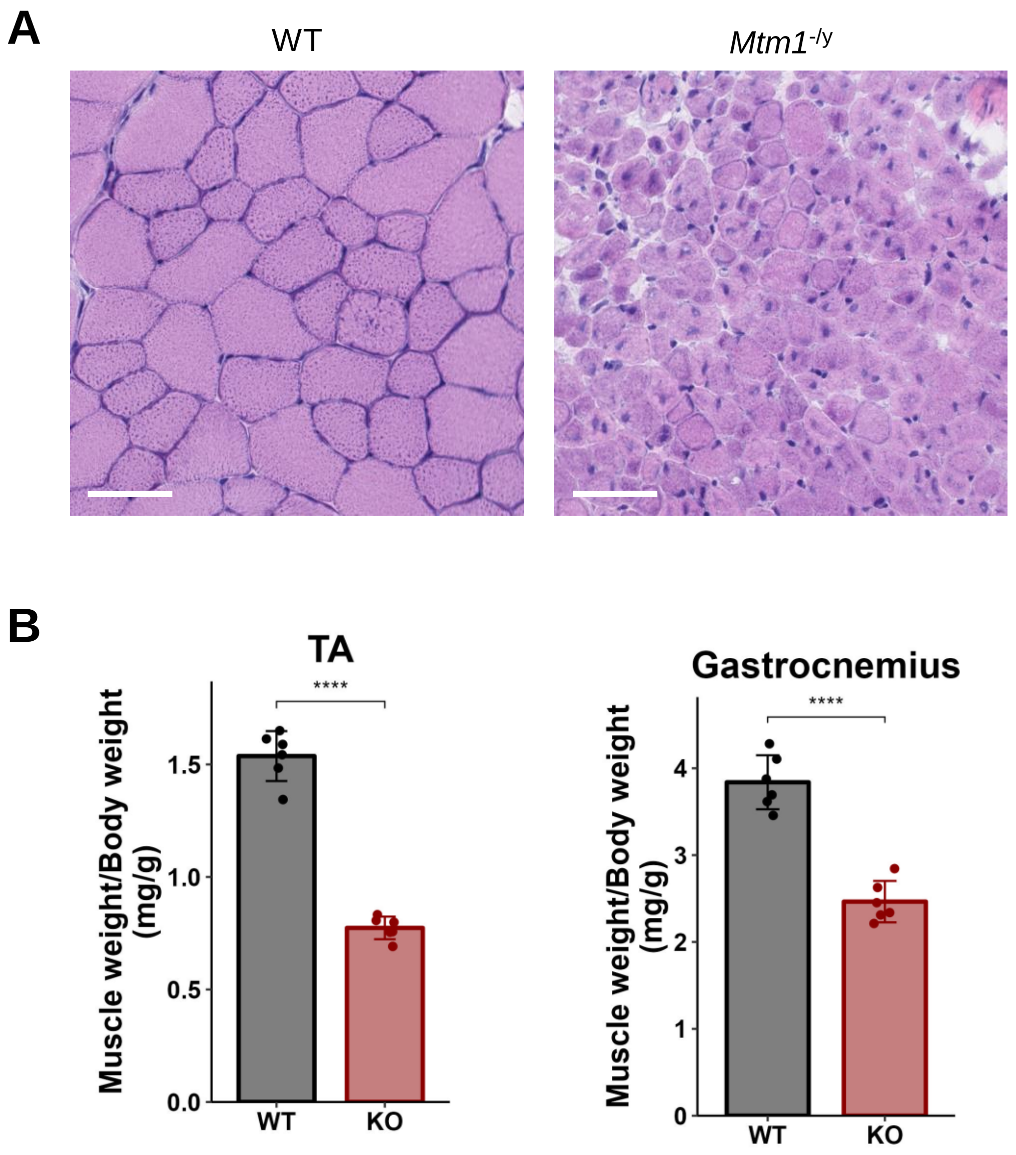

Figure S4. Skeletal muscle defects in *Mtm1*$^{-/y}$ mice. (A) Representative tibialis anterior histology of WT (left) and *Mtm1*$^{-/y}$ (right) mice at 5 weeks (H&E). Scale bars 50µm. **(B)** Bar plot showing tibialis anterior (left) and gastrocnemius (right) weights relative to body weight in WT and *Mtm1*$^{-/y}$ mice at 7 weeks. Student's t-test: ns: P>0.05, *: P < 0.05, **: P < 0.01, ***: P < 0.001, ****: P<0.0001

Figure S5

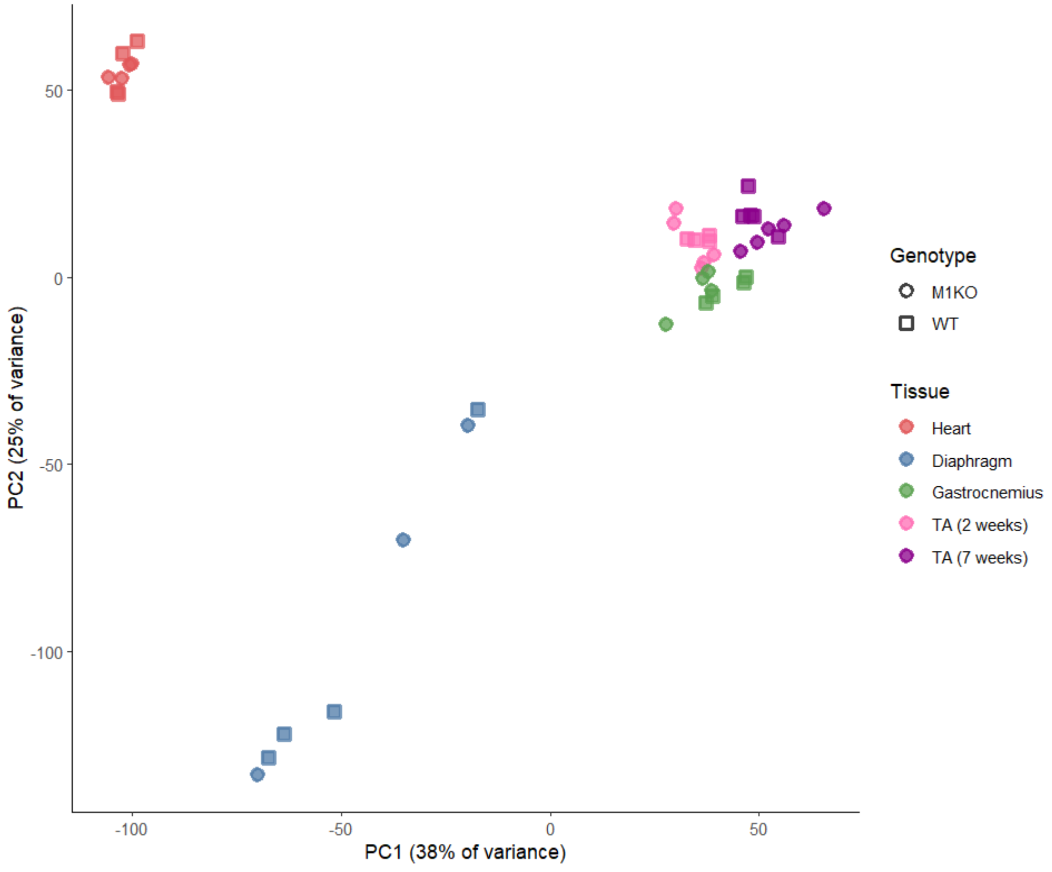

Figure S5. Principal component analysis of heart, gastrocnemius, diaphragm and tibialis anterior samples.

Figure S6

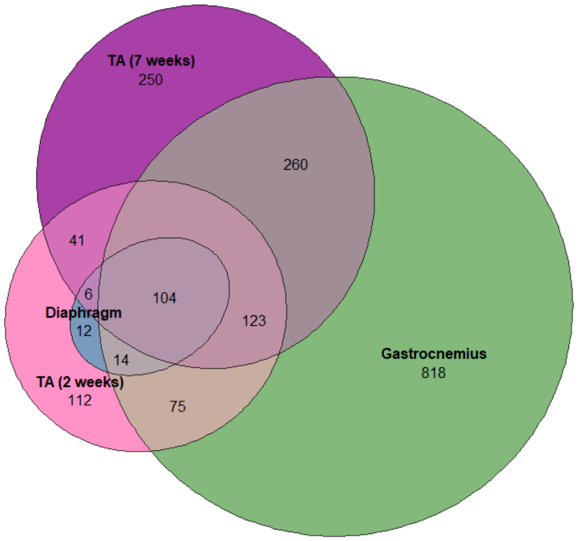

Figure S6. Venn diagram of enriched GO terms in gastrocnemius, diaphragm, tibialis anterior (2 weeks and 7 weeks). GO terms over representation analysis was performed based on the differentially expressed genes between *Mtm1*$^{-/y}$ and WT samples.

Figure S7

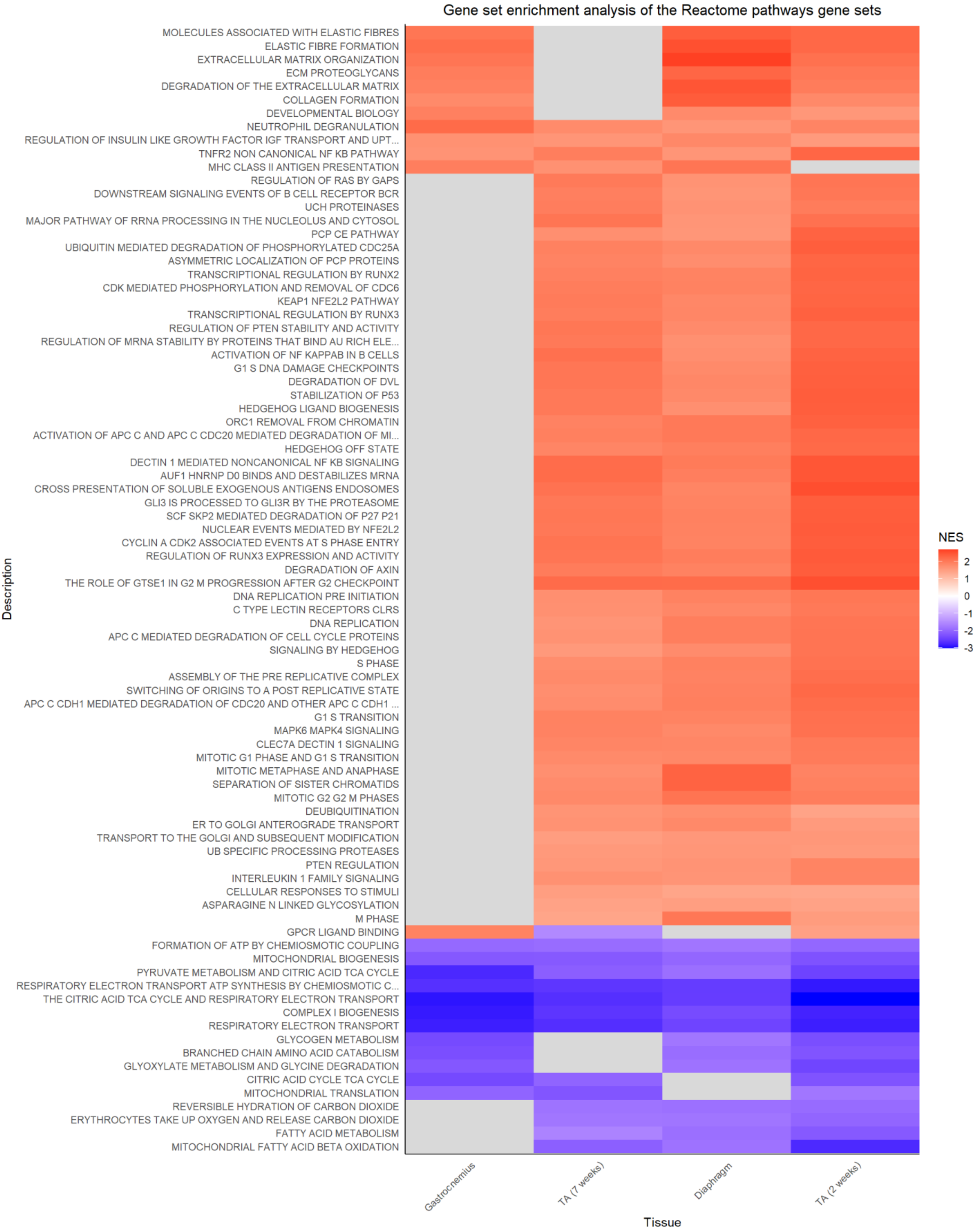

Figure S7. Gene set enrichment analysis results of Reactome pathways gene sets in the skeletal muscles. Red and blue respectively indicate statistically significant positive and negative normalized enrichment scores (NES). Gene sets with significant enrichment in at least three groups are shown.

Figure S8

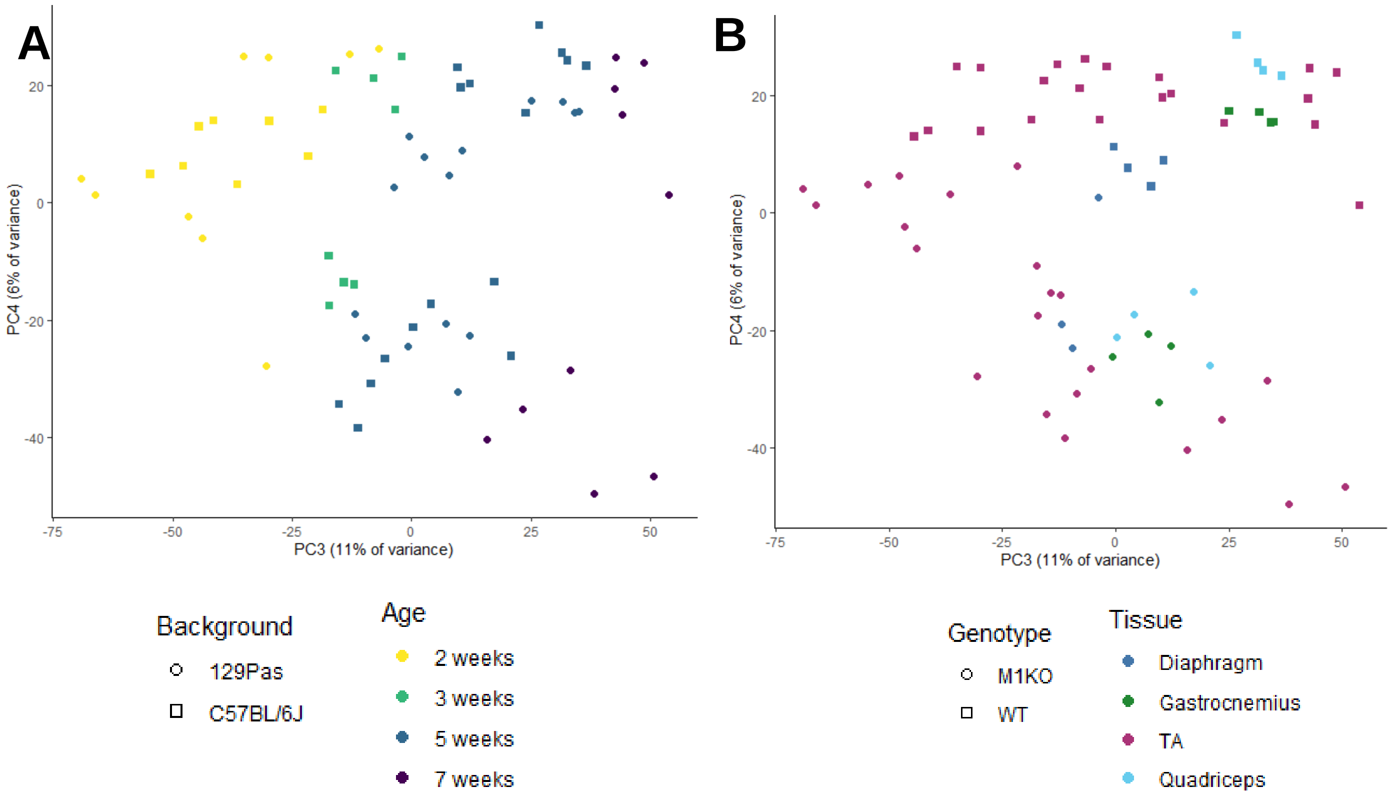

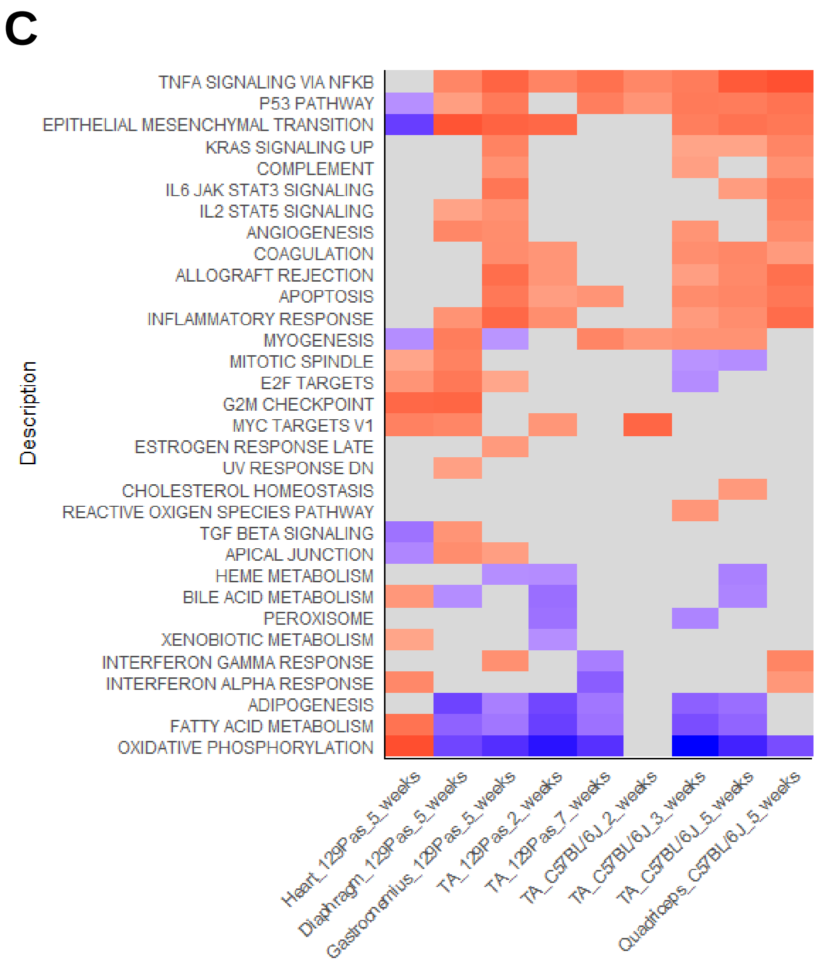

Figure S8. Comparison of RNA-seq datasets obtained from skeletal muscles of 129Pas and C57BL/6J *Mtm1*$^{-/y}$ and WT mice. (A) PCA of diaphragm, gastrocnemius, tibialis anterior and quadriceps samples at several ages. PC 3 and 4 are shown. The shape of the points indicate the genetic background and the color indicates the age. **(B)** Same PCA as (A), with the shape indicating the genotype and the color indicating the tissue **(C)** Gene set enrichment analysis results of MSigDb Hallmarks gene sets in the skeletal muscles. Red and blue respectively indicate statistically significant positive and negative normalized enrichment scores (NES).

Figure S9

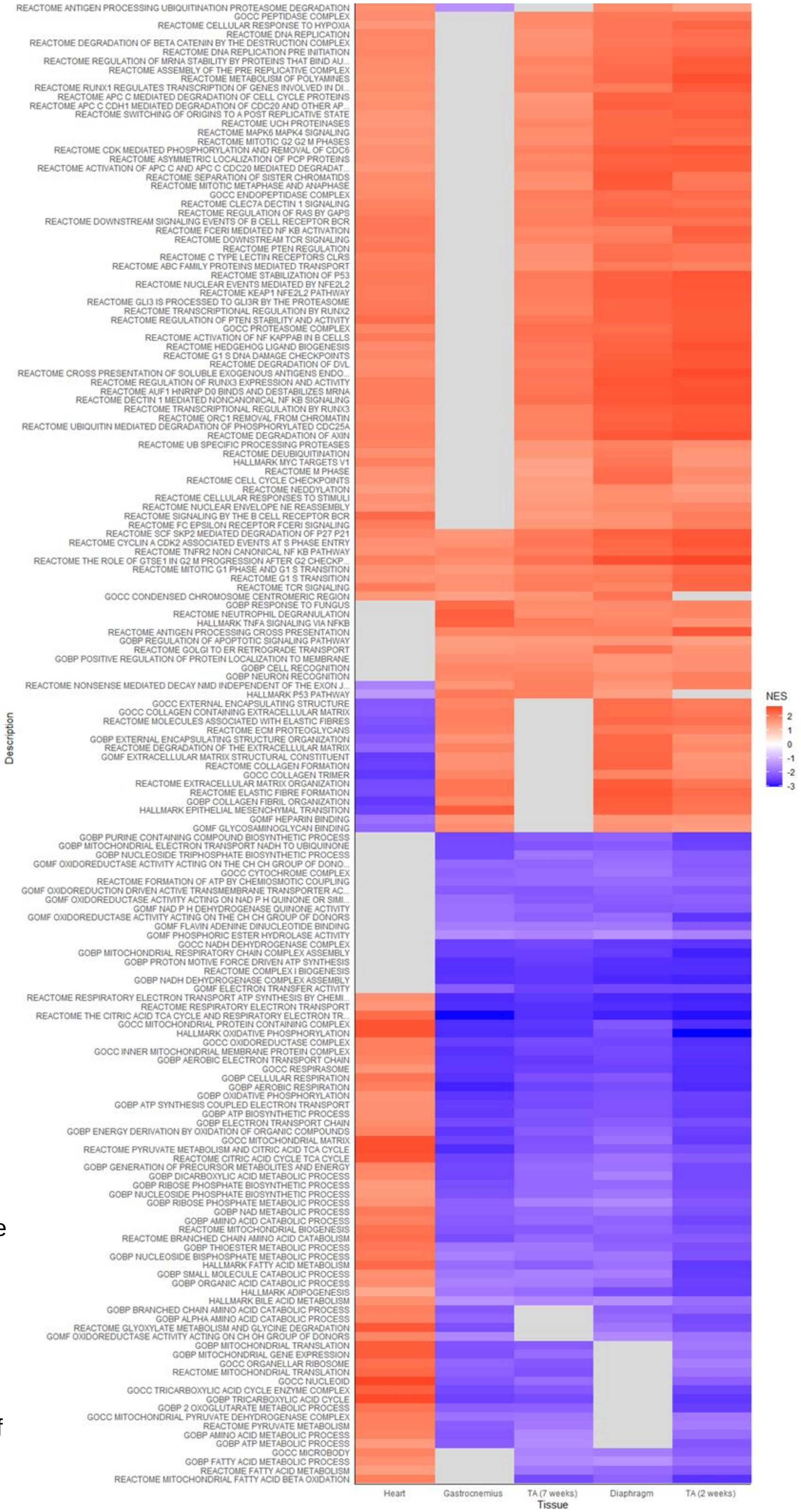

Figure S9. Gene set enrichment analysis results of MSigDb Hallmarks, Reactome pathways and GO terms gene sets in cardiac and skeletal muscles. Red and blue respectively indicate statistically significant positive and negative normalized enrichment scores (NES). Gene sets significantly enriched in at least 4 of the tissues are shown.

Figure S10

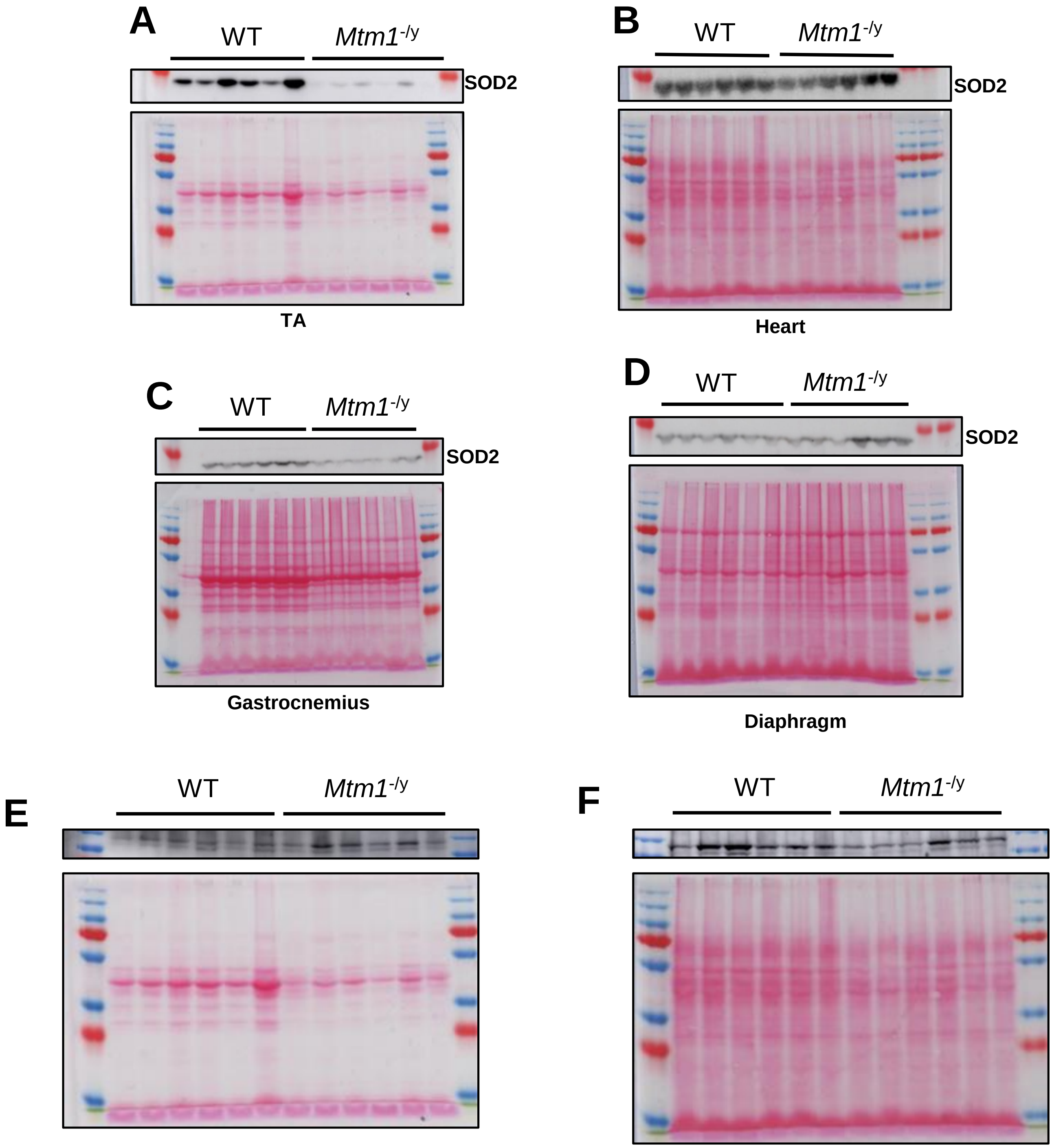

Figure S10. SOD2 and DNM2 Western Blots. Immunostained SOD2 bands (top), Ponceau-stained full blot (bottom) of **(A)** tibialis anterior, **(B)** heart, **(C)** gastrocnemius, and **(D)** diaphragm WT and *Mtm1*$^{-/y}$ samples. Immunostained DNM2 bands (top) and Ponceau-stained full blot (bottom) of **(E)** tibialis anterior and **(F)** heart WT and *Mtm1*$^{-/y}$ samples. Student's t-test: ns: P>0.05, *: $P < 0.05$, **: $P < 0.01$, ***: $P < 0.001$, ****: P<0.0001
